# Enhanced Disease Susceptibility 1 and Salicylic Acid Act Redundantly to Regulate Resistance Gene-Mediated Signaling

**DOI:** 10.1371/journal.pgen.1000545

**Published:** 2009-07-03

**Authors:** Srivathsa C. Venugopal, Rae-Dong Jeong, Mihir K. Mandal, Shifeng Zhu, A. C. Chandra-Shekara, Ye Xia, Matthew Hersh, Arnold J. Stromberg, DuRoy Navarre, Aardra Kachroo, Pradeep Kachroo

**Affiliations:** 1 Department of Plant Pathology, University of Kentucky, Lexington, Kentucky, United States of America; 2 Department of Statistics, University of Kentucky, Lexington, Kentucky, United States of America; 3 United States Department of Agriculture–Agricultural Research Service, Washington State University, Prosser, Washington, United States of America; The University of North Carolina at Chapel Hill, United States of America

## Abstract

Resistance (R) protein–associated pathways are well known to participate in defense against a variety of microbial pathogens. Salicylic acid (SA) and its associated proteinaceous signaling components, including enhanced disease susceptibility 1 (EDS1), non–race-specific disease resistance 1 (NDR1), phytoalexin deficient 4 (PAD4), senescence associated gene 101 (SAG101), and EDS5, have been identified as components of resistance derived from many R proteins. Here, we show that EDS1 and SA fulfill redundant functions in defense signaling mediated by R proteins, which were thought to function independent of EDS1 and/or SA. Simultaneous mutations in EDS1 and the SA–synthesizing enzyme SID2 compromised hypersensitive response and/or resistance mediated by R proteins that contain coiled coil domains at their N-terminal ends. Furthermore, the expression of *R* genes and the associated defense signaling induced in response to a reduction in the level of oleic acid were also suppressed by compromising SA biosynthesis in the *eds1* mutant background. The functional redundancy with SA was specific to EDS1. Results presented here redefine our understanding of the roles of EDS1 and SA in plant defense.

## Author Summary

Salicylic acid and enhanced disease susceptibility 1 are important components of resistance gene-mediated defense signaling against diverse pathogens in a variety of plants. Present understanding of plant defense signaling pathways places salicylic acid and enhanced disease susceptibility 1 downstream of resistant protein activation. In addition, enhanced disease susceptibility 1 is primarily thought to function in the signaling initiated via Toll-interleukin 1-receptor type of resistance proteins. Here, we show that salicylic acid and enhanced disease susceptibility 1 serve redundant functions in defense signaling mediated by coiled-coil-domain containing resistance proteins that were thought to function independent of enhanced disease susceptibility 1. Furthermore, resistance signaling induced under low oleic acid conditions also requires enhanced disease susceptibility 1 and salicylic acid in a redundant manner, but these components are required upstream of resistance gene expression. Together, these results show that the functional redundancy between salicylic acid and enhanced disease susceptibility 1 has precluded their detection as required components of many resistance protein–signaling pathways.

## Introduction

Plants have evolved highly specific mechanisms to resist pathogens. One of the common ways to counter pathogen growth involves the deployment of resistant (R) proteins, which confer protection against specific races of pathogens carrying corresponding avirulence (*Avr*) genes [1]. Following recognition of the pathogen, one or more signal transduction pathways are induced in the host plant and these lead to the prevention of colonization by the pathogen. Induction of defense responses is often accompanied by localized cell death at the site of pathogen entry. This phenomenon, termed the hypersensitive response (HR), is one of the earliest visible manifestations of induced defense reactions and resembles programmed cell death in animals [1]–[6]. Concurrent with HR development, defense reactions are triggered in both local and distant parts of the plant and accompanied by a local and systemic increase in endogenous salicylic acid (SA) levels and the upregulation of a large set of defense genes, including those encoding pathogenesis-related (PR) proteins [7]–[9].

The SA signal transduction pathway plays a key role in plant defense signaling (see reviews in [10]–[12]). Arabidopsis mutants that are impaired in SA responsiveness, such as *npr1* (Nonexpressor of PR; [13]–[15]), or are defective in pathogen-induced SA accumulation, such as *eds1* (Enhanced Disease Susceptibility 1; [16]), *eds5* (Enhanced Disease Susceptibility 5; [17]), *sid2* (isochorishmate synthase; [18]) and *pad4* (Phytoalexin Deficient 4; [19]), exhibit enhanced susceptibility to pathogen infection and show impaired *PR* gene expression. The EDS1, EDS5, PAD4, NPR1 and SID2 proteins participate in both basal disease resistance to virulent pathogens as well as R protein-mediated resistance to avirulent pathogens [20]. Defense signaling mediated via a majority of R proteins, which contain Toll-interleukin1-like (TIR) domains at their N-terminal ends, is dependent on EDS1 [21]. Conversely, the NDR1 (Non-race-specific Disease Resistance) protein is required for many R proteins that contain coiled-coil (CC) domains at their N-terminal ends. However, several CC-nucleotide binding site (NBS)-leucine rich repeat (LRR) type of R proteins, including RPP8, RPP13-Nd, HRT, and RPP7, signal resistance via a pathway(s) that is independent of *NDR1* [21], [22]–[24]. Strikingly, the CC-NBS-LRR gene *HRT*, which confers resistance to Turnip Crinkle Virus (TCV), is dependent on *EDS1* [23]. Besides HRT, the only other CC domain-containing R protein that utilizes an EDS1-dependent pathway is RPW8, which confers broad-spectrum resistance to powdery mildew [25]. However, RPW8 is not a typical NBS-LRR type of R protein; it contains an N-terminal transmembrane domain in addition to the CC domain. Although several components contributing to resistance against pathogens have been identified, the molecular signaling underlying *R* gene-mediated resistance still remains obscure. Furthermore, potential relationship(s) among different downstream components and how they relay information leading to resistance remains unknown.

The EDS1 and PAD4 proteins are structurally related to lipase/esterase-like proteins although their lipase-like biochemical functions have not been demonstrated [16],[19]. EDS1 interacts with PAD4 and SAG (senescence associated gene) 101 and the combined activities of these proteins are required for HR formation and to restrict the growth of virulent bacterial strains [26]. PAD4 and SAG101 also restrict the post-invasive growth of non-pathogenic fungi in Arabidopsis [27].

In addition to the major phytohormone-mediated defense pathways, fatty acid (FA)-derived signals have emerged as important mediators of defense signaling [28]–[35]. The Arabidopsis *SSI2*/*FAB2*-encoded stearoyl-acyl carrier protein-desaturase (SACPD) converts stearic acid (18∶0) to oleic acid (18∶1). A mutation in *SSI2* results in the accumulation of 18∶0 and a reduction in 18∶1 levels. The mutant plants show stunting, spontaneous lesion formation, constitutive *PR* gene expression, and enhanced resistance to bacterial and oomycete pathogens [29],[36]. Characterization of *ssi2* suppressor mutants has shown that the altered defense-related phenotypes are the result of the reduction in the levels of the unsaturated FA, 18∶1 [30], [31], [35], [37]–[40]. The altered defense-related phenotypes in *ssi2* plants can be rescued by restoring the 18∶1 levels via second site mutations in genes encoding a glycerol-3-phosphate (G3P) acyltransferase [*ACT1*, 30], a G3P dehydrogenase [*GLY1*, 31], and an acyl carrier protein [*ACP4*, 35]. A mutation in *act1* disrupts the acylation of G3P with 18∶1 resulting in the increased accumulation of 18∶1, thereby restoring wild-type (wt) phenotypes in *ssi2* plants. ACT1 preferentially utilizes 18∶1 conjugated to the ACP4 isoform in Arabidopsis [35]. Thus, a mutation in *acp4* produces similar phenotypes as the *act1* mutant and suppresses *ssi2*-mediated signaling by increasing 18∶1 levels [35]. A mutation in *GLY1* also restores 18∶1 levels in *ssi2 gly1* plants because it disrupts the formation of G3P from dihydroxyacetone phosphate [31]. Reduced availability of G3P in turn impairs the ACT1-catalyzed reaction resulting in accumulation of 18∶1 in *ssi2 gly1* plants. Concurrently, increasing the endogenous G3P levels via exogenous application of glycerol reduces 18∶1 levels and induces *ssi2*-like phenotypes in wt plants [31],[40]. This effect of glycerol is highly specific because *ssi2*-associated phenotypes are not induced upon glycerol treatment of *act1* (defective in the acylation of G3P with 18∶1) or *gli1* (defective in the phosphorylation of glycerol to G3P) mutants [40].

Recently, we showed that a reduction in 18∶1 levels upregulates the expression of several *R* genes in an SA-independent manner [37]. Furthermore, we showed that pathogen resistance induced via this mode bypasses the requirement for components that are normally required for signaling downstream of R protein activation. For example, resistance to TCV mediated by the *R* gene *HRT* (HR to TCV), requires the recessive locus *rrt* (regulates resistance to TCV), SA, *EDS1* and *PAD4* [23]. Exogenous application of SA induces the expression of *HRT* and overcomes the requirement for *rrt*. However, exogenous SA is unable to induce *HRT* or confer resistance in *pad4* background [23]. Interestingly, even though a reduction in 18∶1 levels also upregulates *HRT* expression to confer resistance to TCV, this mode of resistance is independent of *PAD4*, SA, *EDS1* and *EDS5*, which are required for *HRT*-mediated resistance to TCV [37]. Remarkably, induction of *R* genes in response to reduced 18∶1 is conserved in plants as diverse as Arabidopsis and soybean [41]. Furthermore, this low 18∶1-mediated induction of defense responses was also demonstrated in rice recently [42]. Together, these studies strengthen the conserved role of 18∶1 in plant defense signaling.

Here, we show that *R* gene expression induced in response to a reduction in 18∶1 levels and the associated defense signaling can be suppressed by simultaneous mutations in *EDS1* and the genes governing synthesis of SA. We also show that EDS1 and SA function redundantly in *R* gene-mediated resistance against bacterial, viral and oomycete pathogens and that EDS1 also regulates signaling mediated by CC domain containing R proteins.

## Results

### EDS1 and SA are essential but redundant components required for *R* gene expression induced in response to a reduction in 18∶1 levels

**Figure 1:**
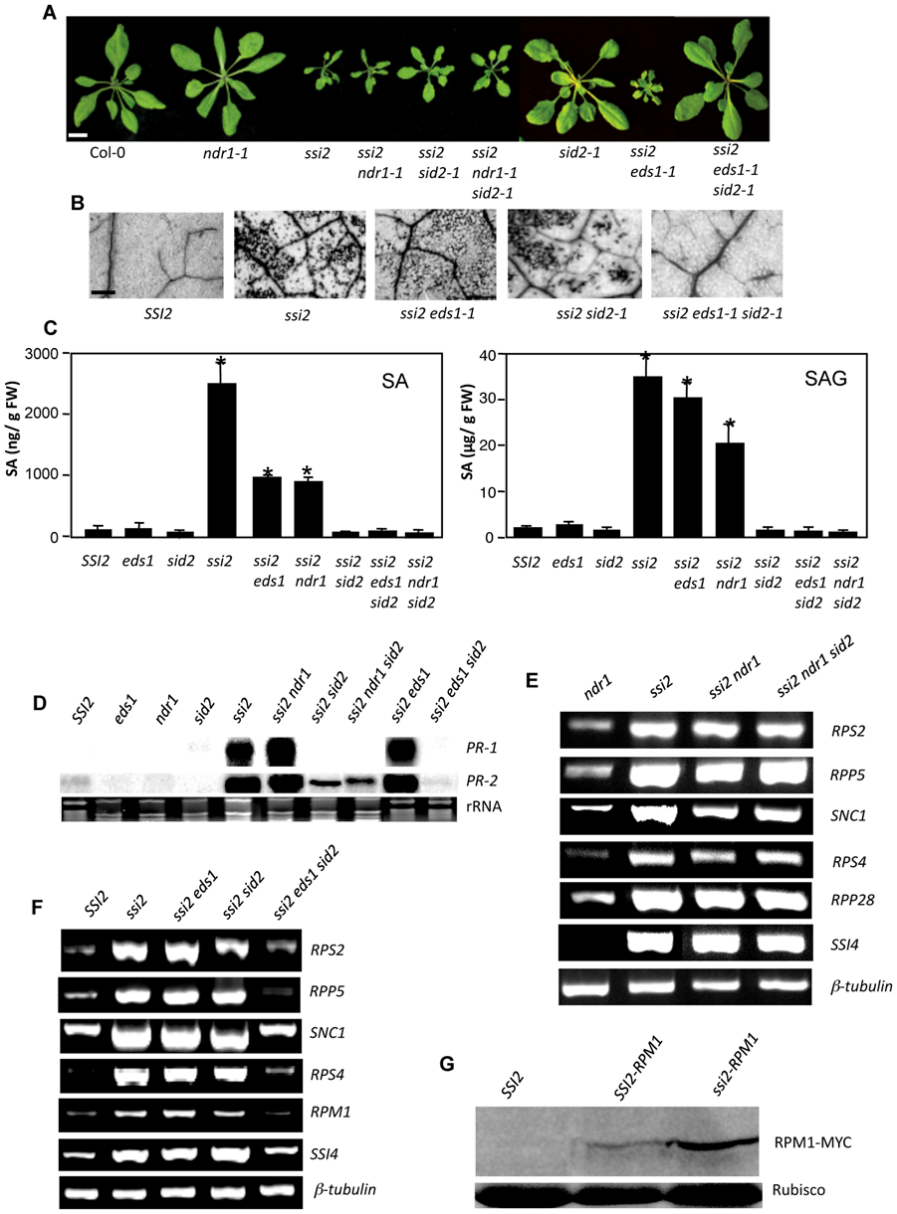
Morphological, molecular, and defense phenotypes of *ssi2 ndr1-1 sid2-1* and *ssi2 eds1-1 sid2-1* plants. (A) Comparison of the morphological phenotypes displayed by 3-week-old soil-grown plants (scale, 0.5 cm). (B) Microscopy of trypan blue-stained leaves from wt (*SSI2*, Col-0 ecotype), *ssi2*, *ssi2 eds1-1*, *ssi2 sid2-1* and *ssi2 eds1-1 sid2-1* plants (scale bars, 270 microns). (C) SA and SAG levels in indicated genotypes. The error bars indicate SD. Asterisks indicate data statistically significant from wt Nö ecotype (*SSI2*) (P<0.05, n = 4). (D) Expression of *PR-1* and *PR-2* genes in indicated genotypes. Total RNA was extracted from 4-week-old plants and used for RNA gel-blot analysis. Ethidium bromide staining of rRNA was used as the loading control. The *PR-1* transcript levels in *EDS1 SID2* F2 plants were similar to those of wt plants (data not shown). (E) RT-PCR analysis of various *R* genes in indicated genotypes. The level of *β-tubulin* was used as an internal control to normalize the amount of cDNA template. (F) RT-PCR analysis of various *R* genes in indicated genotypes. The level of *β-tubulin* was used as an internal control to normalize the amount of cDNA template. The expression of *R* genes in *EDS1 SID2* F2 plants was similar to that of wt plants (data not shown). (G) Levels of Myc-tagged RPM1 protein in indicated genotypes. Levels of Rubisco were used as the loading control.

Signaling mediated by many *R* genes is known to require EDS1 and/or NDR1. Previously, we have shown that *ssi2 eds1* plants continue to express *R* genes at high levels, including those that are dependent on *EDS1* for their signaling [37]. To determine if *NDR1* played a role in *ssi2*-triggered phenotypes, we generated *ssi2 ndr1* plants. The double-recessive plants segregated in a Mendelian fashion and all *ssi2 ndr1* plants showed *ssi2*-like morphology in the F2, F3 and F4 generations (Figure 1A; Table S1). Although the *ssi2 ndr1* plants accumulated significantly less SA/SAG (Figure 1C), compared to *ssi2* plants, they showed *ssi2*-like *PR-1* and *R* gene expression (Figure 1D and 1E, Figure S1A). Exogenous glycerol application, which reduces 18∶1 levels, also induced *R* gene expression in *eds1* and *ndr1* plants (data not shown). Together, these results suggest that *R* gene expression induced by low 18∶1 levels does not require *EDS1* or *NDR1*.

The SA/SAG levels in *ssi2 eds1* and *ssi2 ndr1* plants were significantly higher compared to those in wt plants (Figure 1C). To determine whether high SA in these genotypes was responsible for increased *R* gene expression, we generated *ssi2 eds1 sid2* and *ssi2 ndr1 sid2* plants. Interestingly, only the *ssi2 eds1 sid2* plants showed wt-like morphology and did not develop visible or microscopic cell death (Figure 1A and 1B). In contrast, *ssi2 sid2*, *ssi2 ndr1*, *ssi2 ndr1 sid2* or *ssi2 eds1* plants exhibited *ssi2*-like phenotypes. *PR-1* gene expression was restored to wt-like levels in the *ssi2 eds1 sid2* and *ssi2 ndr1 sid2* plants, due to the *sid2*-derived reduction in SA levels (Figure 1D). In contrast, expression of the SA-independent *PR-2* gene was restored to basal levels only in *ssi2 eds1 sid2* [43], but not in *ssi2 sid2* or *ssi2 ndr1 sid2* plants (Figure 1D, Table S2). Most importantly, *ssi2 eds1 sid2* showed basal expression of *R* genes, unlike *ssi2 ndr1 sid2* plants (Figure 1E and 1F; Figure S1A, S1B; Table S1). *R* gene induction was further confirmed by comparing the transcript profiles of 162 NBS-LRR genes in *ssi2 sid2* with that of wt plants using Affymetrix ATH1 GeneChips arrays. Twenty-one NB-LRR genes were specifically expressed at 2-fold or higher levels in *ssi2 sid2* plants as compared to wt (Col-0) or *eds1* plants (P<0.05) (Table S2). All 21 NB-LRR genes were expressed at low levels in *ssi2 eds1 sid2* plants, further confirming the results from the RT-PCR analysis. Transcriptional profiling performed using Affymetrix arrays showed that the induction of several *R* genes (*RPM1*, *RPS2*, *RPP5*, *RPS4*) was lower than 2-fold in *ssi2* or *ssi2 sid2* compared to wt plants (Table S2, data not shown for *ssi2*). To determine if this low-level induction translated to a significant increase in R protein levels, we analyzed the levels of RPM1 in *ssi2* plants. Indeed, *ssi2* plants accumulated significantly higher levels of the RPM1-Myc protein (Figure 1G).

To rule out the effects of the varied ecotypes of the *ssi2 sid2 eds1* (Nössen, Col-0, Ler) plants we introduced *eds1-1* (Ws-0 ecotype) and *eds1-2* (L*er* ecotype) alleles in *ssi2 sid2* and *ssi2 nahG* (Nössen ecotype) backgrounds (Table S1). All combinations of *ssi2* with *eds1-1/eds1-2* and *sid2/nahG* produced similar phenotypes (data not shown). FA profiling showed that the *ssi2 eds1 sid2* plants contained low 18∶1 levels, similar to *ssi2* plants (Table S3). We thus concluded that *EDS1* and SA function downstream of 18∶1 levels, but upstream of *R* gene expression. Furthermore, *ssi2 eds1 sid2* plants were wt-like, even though neither *ssi2 eds1* nor *ssi2 sid2* were restored for defense signaling. Therefore, EDS1 and SA likely fulfill redundant functions in defense signaling induced in response to a reduction in 18∶1 levels.

**Figure 2:**
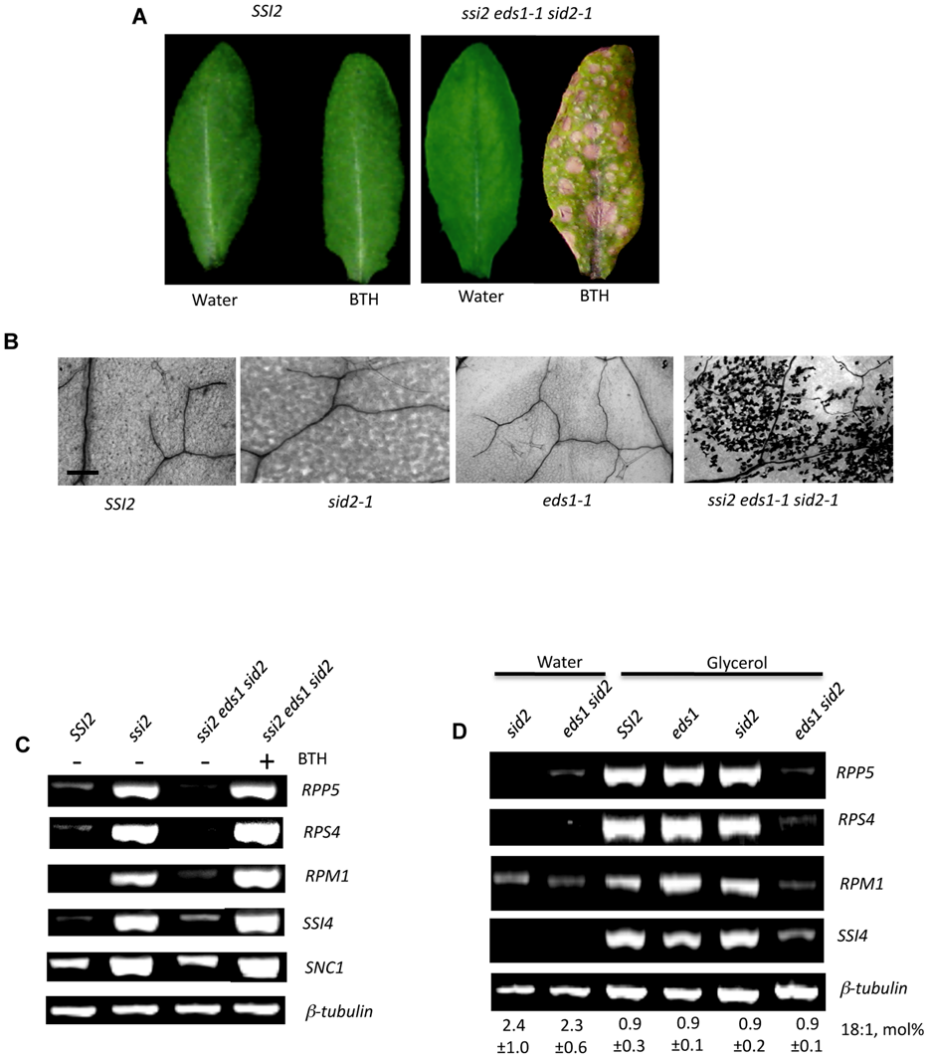
Restoration of *ssi2* phenotypes in *ssi2 eds1-1 sid2-1* plants and glycerol responsiveness of *eds1-1 sid2-1* plants. (A) Visual phenotypes of water- or BTH–treated wt (*SSI2*; Col-0 ecotype) and *ssi2 eds1-1 sid2-1* plants. The plants were photographed at 2 days post treatment (dpt). (B) Microscopy of trypan blue-stained leaves from BTH–treated wt (*SSI2*; Col-0 ecotype), *sid2*, *eds1-1* and *ssi2 eds1-1 sid2-1* plants. The plants were treated with BTH and stained at 2 dpt (scale bars, 270 microns). (C) RT–PCR analysis of *R* genes in water- or BTH-treated *ssi2 eds1-1 sid2-1* plants. Untreated wt (*SSI2*; Col-0 ecotype) and *ssi2* plants were used as controls. The expression of *R* genes in *EDS1 SID2* F2 plants was similar to that of wt plants (data not shown). The level of β-tubulin was used as an internal control to normalize the amount of cDNA template. (D) RT–PCR analysis of various *R* genes in water- or glycerol-treated *sid2-1* and *eds1-1 sid2-1* plants. The glycerol-treated wt (*SSI2*; Col-0 ecotype) and *eds1-1* were included as additional controls. The expression of *R* genes in water- or glycerol-treated *EDS1 SID2* F2 plants was similar to that of water- or glycerol-treated wt plants, respectively (data not shown). The expression of *R* genes in wt and *eds1-1* plants was similar to that seen in *sid2-1* or *eds1-1 sid2-1* plants. The plants were treated with water or glycerol for three days and analyzed for 18∶1 levels and *R* gene expression. The level of β-tubulin was used as an internal control to normalize the amount of cDNA template. The 18∶1 content of each genotype is shown as mol%±SD.

To further test the redundancy for EDS1 and SA, *ssi2 eds1 sid2* plants were treated with SA or its active analog benzo(1,2,3)thiadiazole-7-carbothioic acid (BTH). Application of SA or BTH induced lesion formation on *ssi2 eds1 sid2* plants but not on wt, *eds1*, *sid2*, *eds1 sid2* or *EDS1 SID2* F2 plants (Figure 2A and 2B, data not shown for *eds1 sid2* and *EDS1 SID2*). Also, application of SA or BTH induced *R* gene expression in *ssi2 eds1 sid2* plants (Figure 2C). Thus, application of SA restored *ssi2*-like phenotypes in *ssi2 eds1 sid2* plants. Since glycerol application mimics the effects of the *ssi2* mutation, we generated *eds1 sid2* plants and evaluated them for their ability to induce *R* genes in response to glycerol. Exogenous application of glycerol lowered 18∶1 levels in all genotypes, but induced the expression of *R* genes only in wt, *eds1*, *sid2* and *EDS1 SID2* F2 plants (Figure 2D, Figure S1C). Only a marginal or no increase in *R* gene expression was observed in the *eds1 sid2* plants (Figure 2D). These results confirmed that *EDS1* and SA function redundantly downstream of signaling induced by low 18∶1 levels, but upstream of *R* gene expression.

### EDS1 and SA function redundantly in pathogen resistance induced in response to reduction in 18∶1 levels

**Figure 3:**
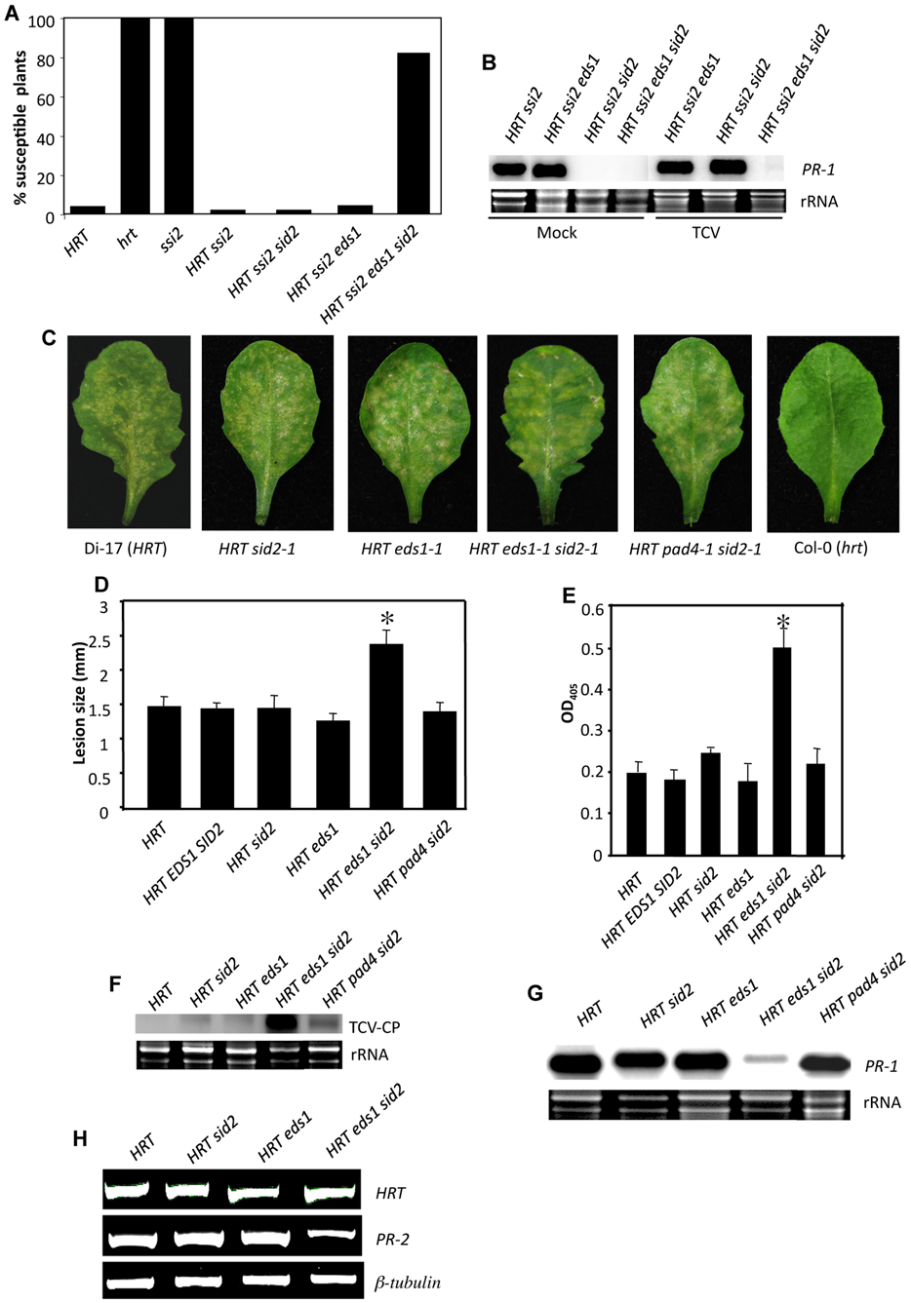
Interaction phenotypes of TCV with *HRT ssi2 eds1-1 sid2-1* and *HRT eds1-1 sid2-1* plants. (A) Percentage TCV susceptible plants. *HRT* and *hrt* indicate resistant and susceptible ecotypes Di-17 and Col-0, respectively. Approximately 70–100 plants were scored for each genotype three-weeks post inoculation and all susceptible plants showed crinkling phenotype and drooping of the bolt [23]. (B) Expression of *PR-1* gene in indicated genotypes after mock- or TCV-inoculation. Total RNA was extracted from inoculated leaves at 3 dpi. Ethidium bromide staining of rRNA was used as the loading control. (C) HR formation in indicated genotypes at 3 dpi. The HR response in TCV-inoculated *HRT EDS1 SID2* F2 plants was similar to that seen in TCV-inoculated Di-17, *HRT sid2-1* or *HRT eds1-1* plants. Plants lacking *HRT* (Col-0, Nö ecotypes or *EDS1 SID2* F2's) did not show any HR. (D) Lesion size in indicated genotypes at 3 dpi. Lesion size was determined from ∼23 individual leaves from each genotype. Statistical significance was determined using Students *t*-test. Asterisks indicate data statistically significant from those of *HRT*, *HRT EDS1 SID2*, *HRT sid2-1* or *HRT eds1-1* plants (P<0.05, n = 23). The error bars indicate SD. (E) ELISA showing levels of TCV CP in the inoculated leaves of indicated genotypes at 3 dpi. Asterisks indicate data statistically significant from results for *HRT* (Di-17 ecotype) plants (P<0.05, n = 4). The error bars indicate SD. (F) Transcript levels of TCV CP in the inoculated leaves of indicated genotypes at 3 dpi. Ethidium bromide staining of rRNA was used as the loading control. (G) Expression of *PR-1* gene in indicated genotypes. Total RNA was extracted from inoculated leaves at 3 dpi. Ethidium bromide staining of rRNA was used as the loading control. The *PR-1* gene expression in TCV-inoculated *HRT EDS1 SID2* F2 plants was similar to that observed in TCV-inoculated *HRT*, *HRT eds1-1* or *HRT sid2-1* plants (data not shown). (H) RT–PCR analysis showing *HRT* and *PR-2* transcript levels in indicated genotypes. The plants were inoculated with TCV and leaf samples were harvested 24 h post inoculation. The level of β-tubulin was used as an internal control to normalize the amount of cDNA template.

We next evaluated the effect of simultaneous mutations in *EDS1*- and SA-signaling pathways on resistance to TCV in the *ssi2* background. We reported previously that resistance to TCV is dependent on the *R* gene, *HRT*, and a recessive locus *rrt* [23]. However, the *ssi2* mutation overcomes the requirement for *rrt* in *HRT*-containing plants [23],[37]. Furthermore, the *ssi2* mutation only confers resistance to TCV when *HRT* is present (Figure 3A). The *ssi2* mutation also overrides a requirement for EDS1 and SA and consequently *ssi2 HRT eds1* as well as *ssi2 HRT sid2* plants exhibit resistance to TCV [37] (Figure 3A). Unlike *HRT ssi2*, *HRT ssi2 eds1* or *HRT ssi2 sid2* plants, the *HRT ssi2 eds1 sid2* plants showed susceptibility to TCV; ∼85% *HRT ssi2 eds1 sid2* plants were susceptible to TCV as against ∼2–4% of *HRT ssi2 sid2* or *HRT ssi2 eds1* plants (Figure 3A). TCV-induced expression of *PR-1* is also independent of *EDS1* and SA. However, TCV inoculation failed to induce *PR-1* expression in *HRT ssi2 eds1 sid2* plants, unlike in *HRT ssi2 sid2* plants (Figure 3B). These results showed that both EDS1 and SA have redundant functions in *ssi2*-mediated resistance to TCV in *HRT* plants.

### EDS1 and SA function redundantly in signaling mediated by *HRT*, *RPS2*, and *RPP8* genes that encode CC-NBS-LRR proteins

To determine the redundancy of EDS1 and SA in signaling mediated by CC-NBS-LRR R proteins, we tested the effects of mutations in EDS1- and/or SID2 on HR to TCV. Earlier, we showed that *HRT*-mediated HR to TCV and *PR-1* gene expression is not affected by mutations in the *EDS1* or *SID2* genes [23]. Consistent with previous results, Di-17 (*HRT*-containing resistant ecotype), *HRT sid2* and *HRT eds1* plants revealed discrete and similar-sized HR lesions on TCV-inoculated leaves (Figure 3C and 3D). In comparison, HR in *HRT eds1 sid2* plants was diffused and formed larger lesions (Figure 3C and 3D). Increased lesion size in *HRT eds1 sid2* plants correlated with increased accumulation of the TCV coat protein (CP) and TCV CP transcript (Figure 3E and 3F). Analysis of *PR-1* and *PR-2* gene expression indicated that TCV-inoculated *HRT eds1 sid2* plants accumulated lower levels of *PR-1* and *PR-2* transcripts, unlike Di-17, *HRT eds1* or *HRT sid2* plants (Figure 3G and 3H). In contrast to *PR*, *HRT* expression remained unaltered in *HRT eds1 sid2* plants (Figure 3H). Together, these results suggested that EDS1 and SA function redundantly in *HRT*-mediated signaling leading to HR formation and expression of *PR-1*. The functional redundancy with SA was specific to EDS1 and did not extend to PAD4; *HRT pad4 sid2* plants showed normal replication of the virus and wt-like HR and *PR-1* gene expression (Figure 3C–3G).

**Figure 4:**
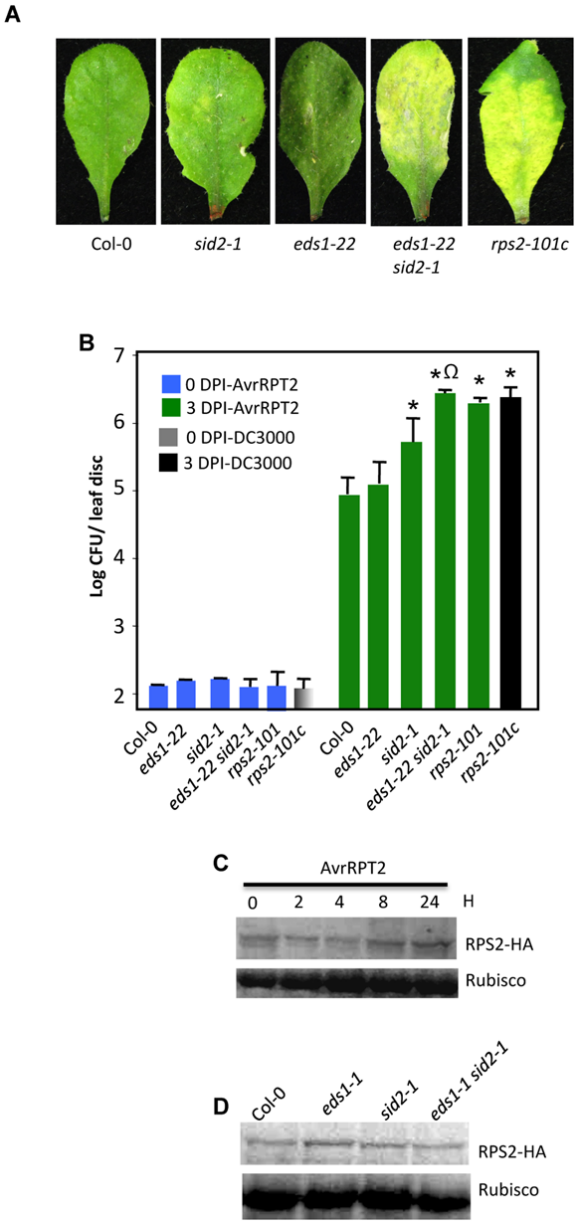
Interaction phenotypes of virulent or *AvrRPT2*-expressing *P. syringae* with *eds1 sid2* plants. (A) Photograph showing phenotypes produced upon infiltration of 10^5^ CFU/ml bacteria (*AvrRPT2*). All genotypes were in the Col-0 background. The leaves were photographed at 3 days post inoculation (dpi). The pathogen-inoculated *EDS1 SID2* F2 plants showed absence of any visible symptoms in response to bacterial inoculations, similar to Col-0 plants (data not shown). (B) Growth of virulent or avirulent (expressing *AvrRPT2*) *P. syrinage* on indicated genotypes. The error bars indicate SD. Asterisks and omega symbols indicate data statistically significant from wt (Col-0) or *sid2* (P<0.05, n = 4), respectively. All genotypes are in the Col-0 background. (C) Levels of HA-tagged RPS2 protein at 0, 2, 4, 8, and 24 h post inoculation with *P. syringae* expressing *AvrRPT2*. Levels of Rubisco were used as the loading control. (D) Levels of HA-tagged RPS2 protein in indicated genotypes. Levels of Rubisco were used as the loading control.

A majority of CC-domain containing R proteins, including RPS2, have been reported as not requiring EDS1 for resistance signaling [21]. To determine the effect of simultaneous mutations in *EDS1* and *SID2* on *RPS2*-mediated resistance, we compared defense phenotypes produced in single or double mutant plants with that of plants lacking a functional *RPS2* gene. Since different alleles of *RPS2* confer varying levels of resistance to *Pseudomonas syringae* (containing *AvrRPT2*) [44], we screened and isolated an *EDS1* knockout (KO) mutant (designated *eds1-22*) in the Col-0 background and crossed it into the *sid2* background (Col-0 ecotype). Inoculation with *P. syringae* expressing *AvrRPT2* induced severe chlorosis on *eds1-22 sid2* leaves (Figure 4A). Similar results were obtained when *P. syringae* expressing *AvrRPT2* was inoculated into *eds1-1 sid2* double mutant plants (Figure S2A). Interestingly, these phenotypes were very similar to those produced on plants lacking a functional *RPS2* (*rsp2-101c*), while *eds1* and *sid2* showed no or very mild symptoms, respectively (Figure 4A, Figure S2A). The appearance of symptoms correlated with bacterial growth; *eds1-22 sid2* plants and the *rps2* mutant supported maximum growth of the pathogen, followed by *sid2* plants (Figure 4B). Similarly, the *eds1-1 sid2* double mutant plants supported more pathogen growth compared to *eds1-1* or *sid2* plants (data not shown). Together, these data suggest that the simultaneous loss of EDS1- and SA-dependent signals is required to mimic a phenotype produced by the loss of the cognate *R* gene, *RPS2*.

To determine if the loss of both EDS1- and SA-dependent signaling impaired resistance by affecting the RPS2 protein, we analyzed R protein levels in *eds1-1* and *sid2* single and *eds1-1 sid2* double mutant plants. Analysis of RPS2 tagged with HA epitope at various times did not detect any significant changes in RPS2 levels in response to inoculation with *P. syringae* expressing *AvrRPT2* (Figure 4C). Therefore, RPS2 levels in mutant plants were analyzed at only 12 and 24 h post-pathogen inoculation. The RPS2-HA levels in *eds1-1*, *sid2* or *eds1-1 sid2* plants were similar to that in wt plants (Figure 4D). These results suggested that abrogation of resistance in *eds1 sid2* double mutants was not due to a defect in the accumulation of the R protein.

**Figure 5:**
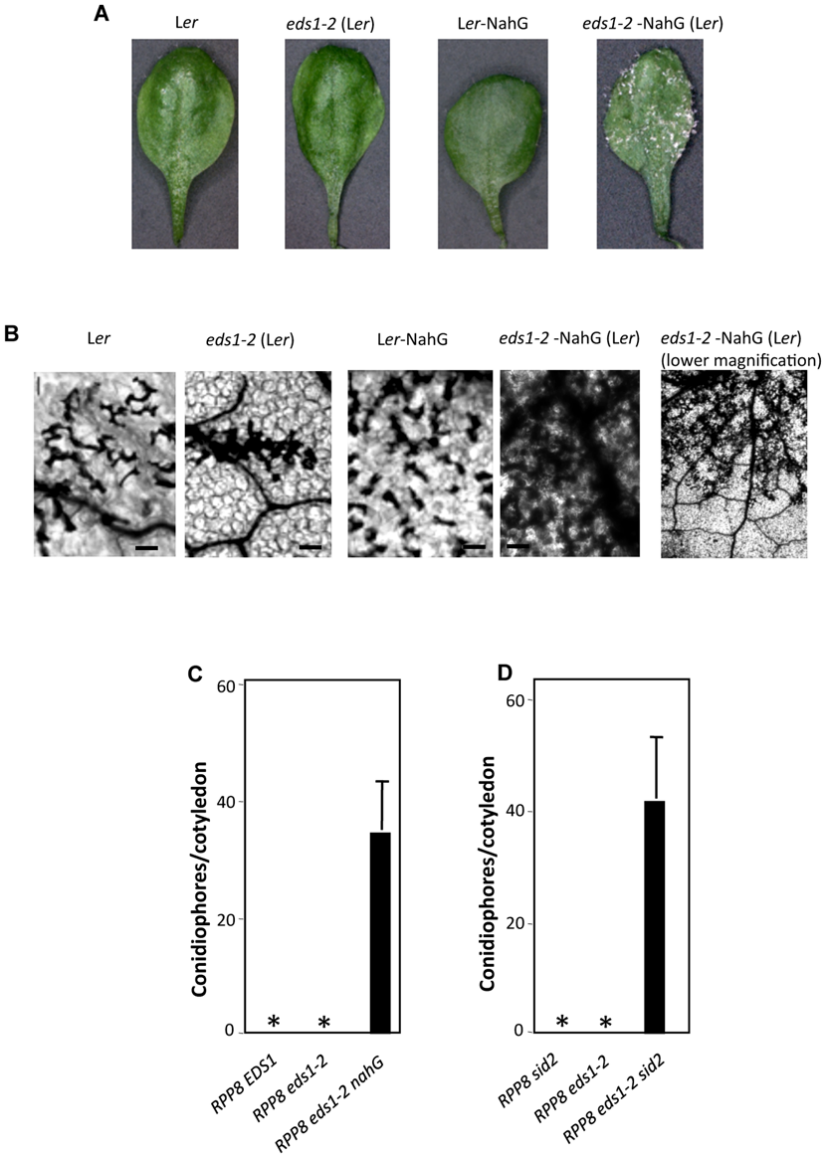
Interaction phenotypes of *H. arabidopsidis* biotype Emco5 expressing *Atr8* with *RPP8 eds1-2 nahG* or *RPP8 eds1-2 sid2-1* plants. (A) Whole leaf pictures showing growth of Emco5 on the cotyledons from indicated genotypes. All genotypes were in the L*er* background. Cotyledons were photographed 10 days after inoculation. (B) Trypan blue stained leaf showing microscopic HR on L*er* and L*er nahG* leaves, and trailing necrosis on *eds1-2* and *eds1-2 nahG* leaves (scale bars, 270 microns). Both high (100×) and low magnification (100×) images of *eds1-2 nahG* leaf are shown. Pathogen inoculations were carried out in F2, F3, and F4 generations with consistent results. The F2 plants showing wt genotype at the mutant locus were resistant to pathogen infection (data not shown). (C) Quantification of pathogen growth on *RPP8 EDS1*, *RPP8 eds1-2* and *RPP8 eds1-2 nahG* plants. Approximately, 40–60 cotyledons were assayed for each genotype. Asterisks indicate absence of spores. All genotypes were in the L*er* background. (D) Quantification of pathogen growth on *RPP8 sid2*, *RPP8 eds1-2*, and *RPP8 eds1-2 sid2-1* plants. All genotypes were in the *ssi2* background. Approximately, 40–60 cotyledons were assayed for each genotype. Asterisks indicate absence of spores.

We next evaluated the effects of mutations in EDS1 and SID2 on *RPP8*-mediated resistance to *Hyalopernospora arabidopsidis* biotype Emco5 encoding *Atr8*. *RPP8* (encodes a CC-NBS-LRR type R protein)-mediated resistance signaling was previously reported to be independent of both *EDS1* and SA [21],[24]. As expected, *RPP8* plants (ecotype L*er*) inoculated with the Emco5 isolate showed localized HR and did not support growth of the pathogen (Figure 5A). Consistent with earlier reports [21],[24], *RPP8 eds1-2* plants also did not support the growth of Emco5, although they did develop trailing necrosis (Figure 5A and 5B). The presence of the *nahG* transgene did not alter HR formation or pathogen response in the *RPP8 nahG* plants (L*er* ecotype). In contrast, *eds1-2 nahG* plants were affected in both HR as well as resistance; *eds1-2 nahG* plants not only showed extensive trailing necrosis but also supported growth and sporulation of the pathogen (Figure 5A–5C). Although *RPP8 EDS1 nahG* and *RPP8 eds1-2 nahG* plants showed contrasting phenotypes (Figure 5A–5C), we still wanted to rule out the possibility that susceptibility of *eds1 nahG* plants was not due to the accumulation of catechol, which is formed upon degradation of SA by NAHG. Estimation of SA levels in Emco5 inoculated *RPP8* (L*er*) plants showed marginal increase in SA and no significant increase in SAG levels compared to mock-inoculated plants (data not shown). This suggests that Emco5 inoculated *nahG* plants are unlikely to show a significant increase in catechol levels. In addition to this, we tested two independent lines of *RPP8 eds1-2 sid2* (in the *ssi2* background) plants and both showed increased susceptibility to Emco5 (Figure 5D). In comparison, *RPP8 eds1-2* or *RPP8 sid2* genotypes did not support any growth or sporulation of the pathogen (Figure 5D). Taken together, these results show that EDS1 and SA have redundant functions in *RPP8*-mediated resistance to *H. arabidopsidis* Emco5.

### Exogenous SA and overexpression of *EDS1* have additive effects on pathogen resistance in wild-type plants

**Figure 6:**
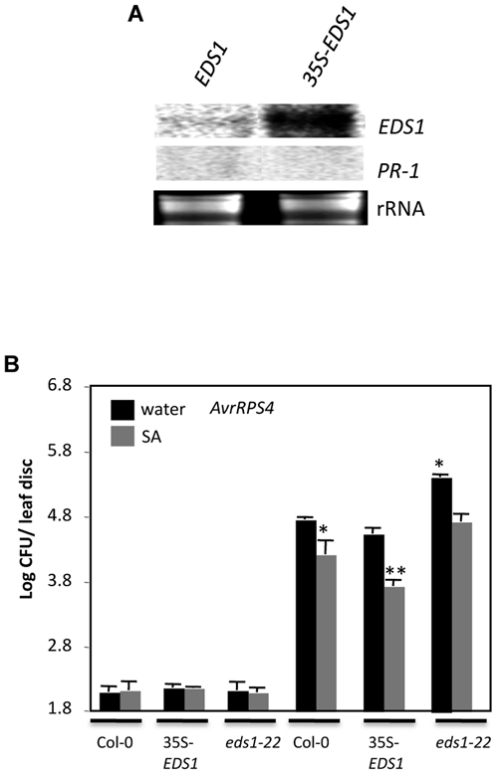
Effect of SA pretreatment and *EDS1* overexpression on pathogen resistance. (A) Expression of *EDS1* and *PR-1* in *EDS1* (Col-0) and 35S-*EDS1* (Col-0) plants. Total RNA was extracted from 4-week-old plants and ethidium bromide staining of rRNA was used as the loading control. (B) Growth of *P. syrinage AvrRPS4* on indicated genotypes (all in Col-0 background). Single asterisks indicate data statistically significant from results for water-treated wt (Col-0) (P<0.05, n = 4). Two asterisks indicate data statistically significant from results for SA–treated wt (Col-0) (P<0.05, n = 4). The error bars indicate SD.

To determine the relation between EDS1- and SA-derived signaling, we compared *PR-1* gene expression and resistance in plants that were either overexpressing *EDS1* or were pretreated with SA. *EDS1* overexpression was achieved by expressing *EDS1* (At3g48090 from the Col-0 ecotype) under control of the CaMV 35S promoter in Col-0 plants (Figure 6A). The 35S-*EDS1* plants analyzed in the T2 and T3 generations showed wt-like morphology (data not shown), wt-like expression of the *PR-1* gene (Figure 6A) and accumulated wt-like levels of SA/SAG (data not shown). In comparison, exogenous application of SA induced *PR-1* and *EDS1* gene expression [data not shown; 16].

Analysis of *RPS4* (encodes a TIR-NBS-LRR type R protein)-mediated resistance showed that exogenous application of SA enhanced resistance to *P. syringae* (expressing *AvrRPS4*) in wt as well as *eds1-22* plants, although wt plants were more resistant to *AvrRPS4* bacteria than the *eds1-22* plants (Figure 6B). Overexpression of *EDS1*, on the other hand, did not alter the response to *AvrRPS4* bacteria. Strikingly, exogenous application of SA on 35S-*EDS1* plants enhanced resistance even more than in the SA-treated wt or *eds1-22* plants. Together, these results suggest that EDS1- and SA-derived signaling contribute additively towards pathogen resistance.

### Simultaneous defects in EDS1 and SA biosynthesis do not additively lower basal defense

**Figure 7:**
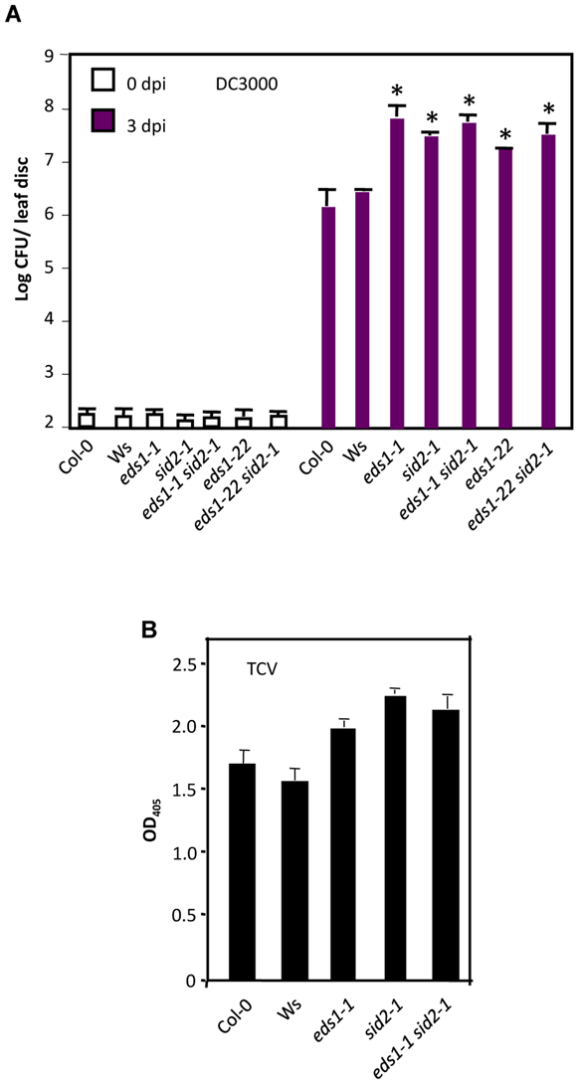
Basal resistance in *eds1 sid2* plants. (A) Growth of virulent *P. syrinage* on indicated genotypes. The error bars indicate SD. Asterisks indicate data statistically significant from wt (Col-0 or Ws) (P<0.05, n = 4). The *eds1-1* and *eds1-22* are in Ws and Col-0 ecotypic backgrounds, respectively. (B) ELISA showing levels of TCV CP in the inoculated leaves of indicated genotypes at 3 dpi. The error bars indicate SD (n = 4).

We next evaluated the effect of the *eds1 sid2* mutations on basal resistance to virulent *P. syringae*, since both EDS1 and SID2 are known to contribute to basal defense as well. The *eds1-1*, *eds1-22*, *sid2* and *eds1 sid2* plants all showed enhanced susceptibility to virulent bacteria as compared to the respective wt ecotypes (Figure 7A). Interestingly, unlike in the case of the avirulent bacteria, growth of virulent bacteria was similar in *eds1 sid2* double mutant plants as compared to that in *eds1* or *sid2* single mutant plants. These results suggested that loss-of-function mutations in *EDS1* and *SID2* do not additively reduce basal resistance to virulent *P. syringae*. Similar to the results obtained with the bacterial pathogen, the loss of both EDS1- and SA-dependent signals did not additively lower basal resistance to TCV either (Figure 7B). This further suggested that the redundant functions of EDS1 and SA might be relevant only for *R* gene-mediated signaling.

### Mutations in *FAD7 FAD8* and *EDS5* restore altered defense signaling in *ssi2 eds1* plants

Besides *SID2*, mutations in *FAD7 FAD8*, which catalyze desaturation of 18∶2 to 18∶3 on membrane glycerolipids, also lower the SA levels in *ssi2* plants [40]. To test if *fad7* or *fad7 fad8* mutations produced a similar effect as *sid2*, these mutations were mobilized into the *ssi2 eds1* background. The *ssi2 eds1 fad7* and *ssi2 eds1 fad7 fad8* plants were bigger in size compared to *ssi2 fad7* or *ssi2 fad7 fad8* plants (Figure S3A). The *ssi2 eds1 fad7 fad8* were wt-like in morphology and showed no or greatly reduced cell death lesions (Figure S3A, S3B). *PR-1* expression was greatly reduced or abolished in *ssi2 eds1 fad7* and *ssi2 eds1 fad7 fad8* plants, respectively (Figure S3C) and correlated with their endogenous SA/SAG levels; the *ssi2 eds1 fad7* and *ssi2 eds1 fad7 fad8* plants showed greatly reduced or basal levels of SA and SAG, respectively (Figure S3D, S3E). Expression of some *R* genes (*SSI4*, *RPS2*, *RPP5*) was nominally or moderately reduced in *ssi2 eds1 fad7* plants (Figure S3D, S3E). By comparison, all *R* genes tested were expressed at basal levels in *ssi2 eds1 fad7 fad8* plants (Figure S3F). These results showed that presence of *fad7 fad8* mutations restored the altered defense phenotypes of *ssi2 eds1* plants. FA profiling did not detect any significant increase in 18∶1 levels in *ssi2 eds1 fad7* and *ssi2 eds1 fad7 fad8* plants, compared to *ssi2 fad7* and *ssi2 fad7 fad8*, respectively (Table S4). This suggested that restoration of defense phenotypes in *ssi2 eds1 fad7 fad8* was not the result of restored 18∶1 levels, but rather the reduction of SA levels in the *eds1* background.

**Figure 8:**
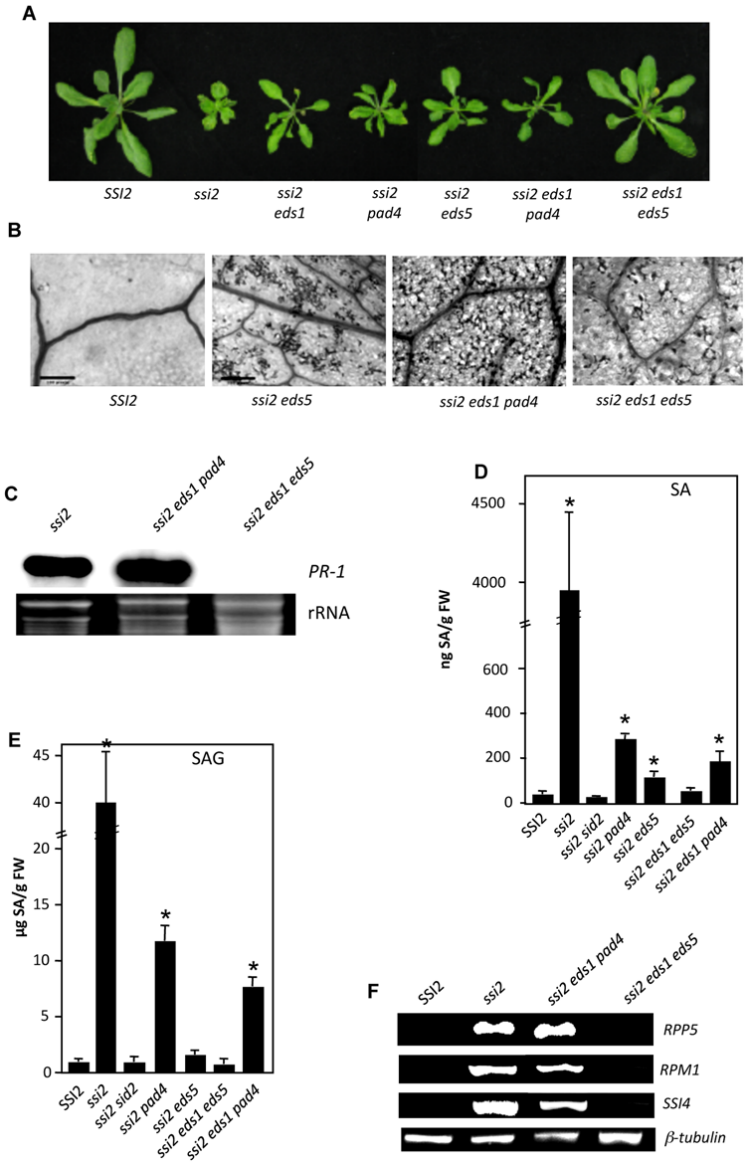
Morphology, cell death, SA/SAG levels. *PR-1* and *R* gene expression *ssi2 eds1-2 pad4-1* and *ssi2 eds1-2 eds5-1* plants. (A) Comparison of the morphological phenotypes displayed by 4-week-old soil-grown wt (*SSI2*), *ssi2*, *ssi2 eds1*, *ssi2 pad4*, *ssi2 eds5*, *ssi2 eds1 pad4*, and *ssi2 eds1 eds5* plants. (B) Microscopy of trypan blue-stained leaves from indicated genotypes. (C) Expression of *PR-1* gene in indicated genotypes. Total RNA was extracted from 4-week-old plants and used for RNA gel-blot analysis. Ethidium bromide staining of rRNA was used as the loading control. (D) Endogenous SA levels in the leaves of 4-week-old soil-grown plants. Values are presented as mean of three replicates and the error bars represent SD. Statistical significance was determined using Students *t*-test. Asterisks indicate data statistically significant compared to *SSI2* (Col-0) plants (P<0.05, n = 5). (E) Endogenous SAG levels in the leaves of 4-week-old soil-grown plants. Values are presented as mean of three replicates and the error bars represent SD. Asterisks indicate data statistically significant compared to *SSI2* (Col-0) plants (P<0.05, n = 5). (F) RT–PCR analysis of *R* genes in indicated genotypes. The level of *β-tubulin* was used as an internal control to normalize the amount of cDNA template. The *SSI2 EDS1*, *SSI2 PAD4*, *SSI2 EDS1 PAD4*, and *SSI2 EDS1 EDS5* F2 plants showed wt–like morphology, accumulated basal levels of SA and showed basal level expression of *PR-1* and *R* genes (data not shown).

Mutations in *EDS5* and *PAD4* also lower SA/SAG levels in *ssi2* plants [40]. To determine if mutations in these can substitute for *sid2* triple mutants containing *ssi2 eds1 pad4* and *ssi2 eds1 eds5* were generated. The *ssi2 eds1 pad4* plants were morphologically similar to *ssi2 eds1* or *ssi2 pad4* plants and showed spontaneous cell death and increased expression of *PR-1* gene (Figure 8A–8C). In comparison, *ssi2 eds1 eds5* showed wt-like morphology, greatly reduced cell death and basal expression of *PR-1* gene (Figure 8A–8C). Quantification of endogenous SA levels showed that both *ssi2 eds1 eds5* and *ssi2 eds1 pad4* accumulated lower SA/SAG levels compared to *ssi2 eds5* and *ssi2 pad4*, respectively (Figure 8D and 8E). However, while *ssi2 eds1 eds5* plants accumulated basal levels of SA/SAG, the *ssi2 eds1 pad4* accumulated significantly higher levels of SA/SAG compared to wt, *ssi2 sid2* and *ssi2 eds1 eds5* plants (Figure 8D and 8E). Analysis of *R* gene expression showed greatly reduced levels in *ssi2 eds1 eds5* plants but the *ssi2 eds1 pad4* expressed *ssi2*-like levels of *R* genes (Figure 8F, Figure S1D). Taken together, these results suggest that the suppression of SA levels was required for the normalization of defense phenotypes in the *ssi2 eds1* background.

### *PAD4*, *SAG101*, and *EDS5* are not functionally redundant with SA in low 18∶1-mediated signaling

Besides *EDS1*, the SA signaling pathway is also regulated by *PAD4* and *EDS5* and via the physical association of EDS1 with SAG101 and PAD4 [17],[19],[45]. To determine if *PAD4*, *SAG101* or *EDS5* also function redundantly with SA, we introduced the *pad4*, *sag101* and *eds5* mutations in the *ssi2* and *ssi2 sid2* backgrounds.

The *ssi2 sag101*, *ssi2 pad4* and *ssi2 eds5* plants showed *ssi2*-like morphology, visible and microscopic cell death and constitutive *PR-1* gene expression (Figure S4A, S4B, S4C and Figure S5A, S5B, S5C). Consistent with these phenotypes, the *ssi2 sag101*, *ssi2 pad4*, *ssi2 eds5* plants showed increased expression of *R* genes (Figure S4D and Figure S5D) and accumulated elevated levels of SA and SAG (Figure S4E, S4F and Figure S5E, S5F). Notably, the SA levels in *ssi2 sag101* plants were ∼6-fold lower than in *ssi2* plants, suggesting that *SAG101* contributed to the accumulation of SA in *ssi2* plants. To determine if the reduced SA in the *sag101* background could restore wt-like phenotypes in *ssi2 eds1* plants, triple mutant *ssi2 eds1 sag101* plants were generated. Although the *ssi2 eds1 sag101* plants accumulated significantly lower levels of SA/SAG (Figure S4E, S4F), these plants were only slightly bigger than *ssi2 eds1* or *ssi2 sid2* plants (Figure S4A), showed spontaneous cell death (Figure S4B) and expressed *PR-1* (Figure S4C) and *R* genes constitutively (Figure S4D). We next analyzed the triple mutant *ssi2 sag101 sid2*, *ssi2 pad4 sid2* and *ssi2 eds5 sid2* plants. All the triple mutants contained wt-like levels of SA and SAG (Figure S4E, S4F and Figure S5E, S5F). The *ssi2 sag101 sid2* plants were morphologically similar to *ssi2* plants, showed spontaneous cell death and expressed *R* genes constitutively (Figure S4A, S4B, S4C, S4D). In comparison, the *ssi2 pad4 sid2* and *ssi2 eds5 sid2* plants were bigger in morphology. However, plants of both genotypes showed cell death (Figure S5A, S5B) and expressed *R* genes constitutively (Figure S5D). Together, these data suggest that the functional redundancy with SA was specific only to EDS1 and did not extend to PAD4, SAG101 or EDS5.

## Discussion

SA is long known as an essential modulator of *R* gene-derived signaling in pathogen defense. Several molecular components, including EDS1, have been identified as essential effectors of SA-derived signaling [23],[26],[45]. Since SA upregulates expression of *EDS1*, both SA and EDS1 are thought to function in a positive feedback loop and EDS1 is widely considered an upstream effector of SA [16],[19],[23],[45]. Recent data has shown that EDS1 signals resistance via both SA-dependent as well as SA-independent pathways [46]. Strikingly, EDS1-dependent but SA-independent branch of EDS1 pathway still requires SA pathway for full expression of resistance [46]. In this study, we have characterized the relationship between EDS1 and SA. We show that the two components act in a redundant, and not necessarily sequential manner to regulate *R* gene expression induced in response to a reduction in the levels of the FA 18∶1. Furthermore, EDS1 and SA also function redundantly in *R* gene-mediated defense against viral, bacterial and oomycete pathogens. It appears that the redundant functions of EDS1 and SA may have prevented their identification as required components for signaling mediated by CC-NBS-LRR R proteins. Indeed, *RPS2*-mediated signaling is fully compromised only in *eds1 sid2* and not in the single mutant plants. Similarly, *HRT*-mediated signaling leading to HR formation and *PR-1* gene expression is only affected in *eds1 sid2* plants, while *eds1* or *sid2* plants behave similar to wt plants. Furthermore, *RPP8*-mediated resistance, which was previously reported not to require EDS1 or SA [21],[24], is compromised in plants lacking both EDS1 and SA. In contrast to their effect on *R* gene-mediated resistance, loss of both EDS1- and SA-dependent signals did not additively lower basal resistance to *P. syringae* or TCV. Together, these data suggests that the redundant functions of EDS1 and SA might be relevant only for *R* gene-mediated signaling.

**Figure 9:**
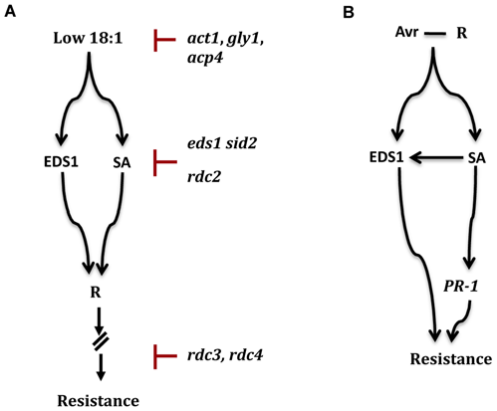
Models for signaling induced by low 18∶1 fatty acid levels and *R* genes. (A) EDS1 and SA function upstream of *R* genes and regulate expression of *R* genes induced by low 18∶1 fatty acid levels. Mutations in *EDS1* and SA-synthesizing enzyme, encoded by *SID2*, abolish constitutive upregulation of *R* genes and associated enhanced resistance in genetic backgrounds containing low 18∶1 levels. Similar to EDS1/SA, restored in defective crosstalk (*RDC*) 2 acts downstream of signaling induced by low levels of 18∶1 but upstream of *R* gene expression. Signaling induced by low 18∶1 fatty acid levels can also be suppressed by mutations in *ACT1*-encoded G3P acyltransferase [30], *GLY1*-encoded G3P dehydrogenase [31], or *ACP4*-encoded acyl carrier protein 4 [35], which normalize 18∶1 levels, or by blocking steps downstream of *R* gene expression (*rdc3* and *rdc4*). Upregulation of *R* genes induced by low 18∶1 fatty acid levels does not require *PAD4*, *SAG101*, or *EDS5*, which are components of the resistance signaling pathway(s) initiated upon R-Avr interaction. (B) Direct or indirect interaction between host-encoded R and pathogen-encoded Avr products initiate resistance signaling, which requires EDS1 and SA. Exogenous application of SA induces expression of *PR-1* and *EDS1* genes but overexpression of *EDS1* does not induce *PR-1* expression or increase SA levels. The EDS1– and SA–dependent pathways have additive effects.

In contrast to SA application, overexpression of *EDS1* was unable to confer increased resistance to the avirulent pathogen *P. syringae*. Furthermore, unlike SA, overexpression of *EDS1* was not associated with the induction of *PR-1* gene expression. These findings, together with the observation that SA was able to induce *EDS1* expression and that SA application on wt plants resulted in higher resistance than that in *eds1*, suggests that SA feedback regulates EDS1-derived signaling in a unidirectional manner (Figure 9B). Thus, SA application induces both SA- and EDS1-derived signaling, the additive effects of which enhance resistance in wt plants much more than in *eds1-22* plants. Furthermore, the combined effects of SA pretreatment and *EDS1* overexpression induced much better resistance than the individual effects of each. This is consistent with a previous report that 35S-*EDS1* plants induce rapid and stronger expression of *PR-1* in response to pathogen inoculation [47]. The additive effects of EDS1 and SA was also supported by the observation that *eds1 sid2* plants showed pronounced chlorosis upon inoculation with *AvrRPS4* expressing pathogen, which is recognized by a TIR-NBS-LRR protein RPS4 (Figure S2B). Since mutations in SA-independent branch of EDS1 pathway and *sid2* have additive effects on *R* gene-mediated resistance [46], it is possible that overexpression of *EDS1* triggers signaling via both SA-dependent and/or -independent branches of EDS1 pathway.

Although the Col-0 ecotype is thought to contain two functional alleles of *EDS1* [26], a KO mutation in At3g48090 was sufficient to compromise both basal and *R* gene (*RPS4*)-mediated resistance. However, the Col-0 *eds1-22* mutant consistently supported less growth of virulent or avirulent pathogens compared to *eds1-1* or *eds1-2* plants. This suggests that the second *EDS1* allele in the Col-0 ecotype might also contribute towards the resistance response. This is consistent with another study where constitutive defense phenotypes due to the overexpression of the *SNC1* gene, encoding a TIR-NBS-LRR R protein, are not completely suppressed by a mutation in *eds1* in the Col-0 background but restored by the *eds1* mutation in the Ws background [48].

The inability to accumulate SA together with a mutation in *EDS1* was also required to suppress constitutive defense signaling resulting from the overexpression of *R* genes induced in response to reduced 18∶1 levels. Although *eds1* or *sid2* plants were entirely competent in inducing *R* gene expression in response to a reduction in 18∶1, *eds1 sid2* plants were not. Thus, *ssi2 eds1 sid2* as well as glycerol-treated *eds1 sid2* plants showed wt-like expression of *R* genes while *ssi2 eds1*, *ssi2 sid2* and glycerol-treated *eds1* or *sid2* plants showed increased expression of *R* genes. Moreover, treatment of *ssi2 eds1 sid2* plants with exogenous SA restored *R* transcript induction and cell death in these plants. The fact that glycerol treatment is unable to induce *R* gene expression in *eds1 sid2* plants supports the possibility that EDS1 and SA function upstream of, and not merely serve as a feedback loop in, *R* gene induction. Signaling induced by low 18∶1 levels continues to function in the absence of SA, suggesting a novel SA-independent role for EDS1 in defense signaling.

Since *ssi2 eds1 sid2* plants contain a mixed ecotypic background (Nö, Ws/L*er*, Col-0, ecotypes), it is possible that ecotypic variations in various genetic backgrounds resulted in the restoration of *ssi2*-triggered defense phenotypes. Indeed, phenotypic variations amongst different Arabidopsis ecotypes have been associated with many physiological processes [48]–[51]. Moreover, certain alleles can express themselves only in specific ecotypic backgrounds [48],[51]. However, since *ssi2 EDS1 SID2*, *ssi2 EDS1 sid2* or *ssi2 eds1 SID2* plants (F2 population) always exhibited *ssi2*-like phenotypes, it is highly unlikely that ecotypic variations resulted in the restoration of phenotypes in *ssi2 eds1 sid2* plants. The effect of ecotypic variations on the observed phenotypes can be further ruled out for the following reasons. First, the effects of different mutations were assessed in multiple backgrounds. For example, we used both *eds1-1* (Ws-0 ecotype) and *eds1-2* (L*er* ecotype) alleles in *ssi2 sid2* (Nö, Col-0 ecotypes) and *ssi2 nahG* (Nö ecotype) backgrounds and all combinations of *ssi2* with *eds1-1/eds1-2* and *sid2/nahG* produced similar phenotypes (Table S1). Second, all defense phenotypes were assessed over three generations using multiple progeny. Third, similar results were obtained when different ecotypic backgrounds were evaluated for their response to different pathogens. For example, *eds1 nahG* or *eds1 sid2* backgrounds conferred increased susceptibility to *H. arabidopsidis*, *P. syringae* and TCV, even though only the genotypes used for TCV were of mixed ecotypic backgrounds. Fourth, F2 plants containing wild-type alleles behaved like wild-type parents. Finally, the effects of various mutant backgrounds on *ssi2* phenotypes were also confirmed by glycerol application on individual mutants.

Although glycerol treatment failed to induce *R* gene expression in *eds1 sid2* plants, it did induce cell death. This is in contrast to the absence of a cell death phenotype in *ssi2 eds1 sid2* leaves. One possibility is that the glycerol-triggered cell death is not due to a reduction in 18∶1 levels. However, significant overlap between *ssi2*- and exogenous glycerol-triggered signaling pathways lessens such a possibility [40]. An alternate possibility is that, while EDS1 affects a majority of the responses induced by low 18∶1 levels, the cell death phenotype is also governed by some additional molecular factor(s). This is supported by the fact that *ssi2 pad4 sid2* plants exhibit improved morphology and reduced cell death even though they are not restored for other defense-related phenotypes.

Since the overexpression of *R* genes can initiate defense signaling in the absence of a pathogen [48],[52], it is possible that the induced defense responses in *ssi2* plants are the result of increased *R* gene expression. This idea is supported by the fact that *ssi2*-related phenotypes can be normalized by restoring *R* gene expression to wt-like levels, irrespective of their 18∶1 levels. Thus, wt-like defense phenotypes are restored in suppressors containing high 18∶1 levels, such as *ssi2 act1*, *ssi2 gly1* or *ssi2 acp4* [30],[31],[35], as well as in suppressor containing low 18∶1 levels, such as *ssi2 eds1 sid2* (this work) and restored in defective crosstalk (*rdc*) 2 (unpublished data) (Figure 9A). We have also characterized additional *ssi2* suppressors that show wt-like phenotypes even though they contain low 18∶1 levels and express *R* genes constitutively (*rdc3*, *rdc4*). Together, these results suggest that the *ssi2*-associated phenotypes can be restored by normalizing *R* gene expression to wt-like levels either by increasing 18∶1 levels, impairing factors downstream of signaling induced by low 18∶1 levels, or impairing events downstream of *R* gene expression induced by low 18∶1 levels.

In addition to 18∶1 levels or *R* gene expression, *ssi2*-related defense signaling could also be normalized by altering some factor(s) that function downstream of *R* gene induction. Indeed, our preliminary characterizations have identified additional *ssi2* suppressors that yield wt-like phenotypes with regards to defense signaling but continue to express *R* genes at high levels. Reduced 18∶1 levels may induce defense signaling by directly regulating the transcription of activators or suppressors of defense gene expression. This is supported by the fact that 18∶1-mediated activation of a transcription factor induces the expression of genes required for neuronal differentiation [53]. Similarly, in *Sacharromyces cerevisiae* as well as mammalian cells, binding of 18∶1 to specific transcription factors induces the transcription of genes carrying 18∶1 responsive elements in their promoters [54],[55]. On the other hand, expression of the oncogene *HER2* is inhibited via the 18∶1-upregulated expression of its transcriptional repressor [56]. Reduced 18∶1 might also directly activate/inhibit/alter protein activities. For example, 18∶1 is known to activate the Arabidopsis phospholipase D [57] and inhibit glucose-6-phosphate transporter activity in *Brassica* embryos [58]. Indeed, we have also identified several Arabidopsis proteins for which enzymatic activities are inhibited upon binding to 18∶1 (unpublished data).

In conclusion, results presented here redefine the currently accepted pathway for SA-mediated signaling by showing that EDS1 and SA play a redundant role in plant defense mediated by R proteins and in signaling induced by low 18∶1 fatty acid levels. Further biochemical characterization should help determine if 18∶1 binds to EDS1 and if cellular levels of 18∶1 modulate the as yet undetected lipase activity of EDS1.

## Materials and Methods

### Plant growth conditions and genetic analysis

Plants were grown in MTPS 144 Conviron (Winnipeg, MB, Canada) walk-in-chambers at 22°C, 65% relative humidity and 14 hour photoperiod. The photon flux density of the day period was 106.9 µmoles m^−2^ s^−1^ and was measured using a digital light meter (Phytotronic Inc, Earth city, MO). All crosses were performed by emasculating the flowers of the recipient genotype and pollinatng with the pollen from the donor. All the genotypes and crosses analyzed in this work, their genetic background and number of single, double, or triple mutant plants studied are listed in Table S1. In most cases, single, double, or triple mutant plants were obtained from more than one combination of crosses and showed similar morphological, molecular and biochemical phenotypes. F2 plants showing the wt genotype at the mutant locus were used as controls in all experiments. The wt and mutant alleles were identified by PCR, CAPS, or dCAPS analysis and/or based on the FA profile [30],[31],[38],[40]. The *EDS1* KO mutant in At3g48090 was, isolated by screening SALK_071051 insertion line, obtained from ABRC. The *EDS1 KO* was designated *eds1-22*, based on the previous designation assigned to SALK_071051 T-DNA KO line [48]. The At3g48090 gene showed 98.8% identity at amino acid level to *EDS1* allele from L*er* ecotype. The homozygous insertion lines were verified by sequencing PCR products obtained with primers specific for the T-DNA left border in combination with an *EDS1*-specific primer. The *eds1-22* lines did not show any detectable expression of *EDS1*.

### RNA extraction and northern analyses

Small-scale extraction of RNA from one or two leaves was performed with the TRIzol reagent (Invitrogen, CA), following the manufacturer's instructions. Northern blot analysis and synthesis of random-primed probes for *PR-1* and *PR-2* were carried out as described previously [29].

### Reverse Transcription–PCR

RNA quality and concentration were determined by gel electrophoresis and determination of A_260_. Reverse transcription (RT) and first strand cDNA synthesis were carried out using Superscript II (Invitrogen, CA). Two-to-three independent RNA preparations were used for RT-PCR and each of these were analyzed at least twice by RT–PCR. The RT–PCR was carried out for 35 cycles in order to determine absolute levels of transcripts. The number of amplification cycles was reduced to 21–25 in order to evaluate and quantify differences among transcript levels before they reached saturation. The amplified products were quantified using ImageQuant TL image analysis software (GE, USA). Gene-specific primers used for RT–PCR analysis are described in Table S5.

### Trypan-blue staining

The leaves were vacuum-infiltrated with trypan-blue stain prepared in 10 mL acidic phenol, 10 mL glycerol, and 20 mL sterile water with 10 mg of trypan blue. The samples were placed in a heated water bath (90°C) for 2 min and incubated at room temperature for 2–12 h. The samples were destained using chloral hydrate (25 g/10 mL sterile water; Sigma), mounted on slides and observed for cell death with a compound microscope. The samples were photographed using an AxioCam camera (Zeiss, Germany) and images were analyzed using Openlab 3.5.2 (Improvision) software.

### Pathogen infections

The asexual conidiospores of *H. arabidopsidis* Emco5 expressing *Atr8* were maintained on the susceptible host Nössen (Nö) or Nö *NahG*. The spores were removed by agitating the infected leaves in water and suspended to a final concentration of 10^5^ spores/mL. Two-week-old seedlings were sprayed with spore suspension and transferred to a MTR30 reach-in chamber (Conviron, Canada) maintained at 17°C, 98% relative humidity and 8 h photoperiod. Plants were scored at ∼10–14 dpi and the conidiophores were counted under a dissecting microscope.

The bacterial strain DC3000 derivatives containing pVSP61 (empty vector), *AvrRpt2* or *AvrRps4* were grown overnight in King's B medium containing rifampicin (Sigma, MO). The bacterial cells were harvested, washed and suspended in10 mM MgCl_2_. The cells were diluted to a final density of 10^5^ to 10^7^ CFU/mL (A_600_) and used for infiltration. The bacterial suspension was injected into the abaxial surface of the leaf using a needle-less syringae. Three leaf discs from the inoculated leaves were collected at 0 and 3 dpi. The leaf discs were homogenized in 10 mM MgCl_2_, diluted 10^3^ or 10^4^ fold and plated on King's B medium.

Transcripts synthesized *in vitro* from a cloned cDNA of TCV using T7 RNA polymerase were used for viral infections [59],[60]. For inoculations, the viral transcript was suspended at a concentration of 0.05 µg/µL in inoculation buffer, and the inoculation was performed as described earlier [56]. After viral inoculations, the plants were transferred to a Conviron MTR30 reach-in chamber maintained at 22°C, 65% relative humidity and 14 hour photoperiod. HR was determined visually three-to-four days post-inoculation (dpi). Resistance and susceptibility was scored at 14 to 21 dpi and confirmed by northern gel blot analysis. Susceptible plants showed stunted growth, crinkling of leaves and drooping of the bolt.

### Transcriptional profiling

Total RNA isolated from four-week-old plants using TRIZOL as outlined above. The experiment was carried out in triplicate and a separate group of plants was used for each set. RNA was processed and hybridized to the Affimetric Arabidopsis ATH1 genome array GeneChip following the manufacturers instructions (http://www.affymetrix.com/Auth/support/downloads/manuals/expression_analysis_technical_manual.pdf). All probe sets on the Genechips were assigned hybridization signal above background using Affymetrix Expression Console Software v1.0 (http://www.affymetrix.com/Auth/support/downloads/manuals/expression_console_userguide.pdf). Data was analyzed by one-way Anova followed by post hoc two sample *t*-tests. The P values were calculated individually and in pair-wise combination for each probe set. The identities of 162 NBS-LRR genes were obtained from the Arabidopsis information resource (TAIR; www.arabidopsis.org) and disease resistance gene homolog databases (http://niblrrs.ucdavis.edu/).

### Fatty acid profiling

FA analysis was carried out as described previously [61]. For FA profiling, one or few leaves of four-week-old plants were placed in 2 ml of 3% H_2_SO_4_ in methanol containing 0.001% butylated hydroxytoluene (BHT). After 30 minutes incubation at 80°C, 1 mL of hexane with 0.001% BHT was added. The hexane phase was then transferred to vials for gas chromatography (GC). One-microliter samples were analyzed by GC on a Varian FAME 0.25 mm×50 m column and quantified with flame ionization detection. The identities of the peaks were determined by comparing the retention times with known FA standards. Mole values were calculated by dividing peak area by molecular weight of the FA.

### SA and SAG quantification

SA and SAG quantifications were carried out from ∼300 mg of leaf tissue as described before [23].

### Chemical treatment of plants

SA treatments were carried out by spraying or subirrigating 3-week-old plants with 500 µM SA or 100 µM BTH. For glycerol treatment, plants were sprayed with 50 mM solution prepared in sterile water.

### Enzyme linked immuno-sorbent assay and western analysis

Total protein was extracted in buffer containing 50 mM Tris pH 8.0, 1 mM EDTA, 12 mM β-mercaptoethanol and 10 µg ml^−1^ phenylmethylsulfonyl fluoride. Proteins were fractionated on a 10–12% SDS-PAGE to confirm the quality. An antigen-coated enzyme-linked immunosorbent assay was used to determine levels of TCV CP in the infected plants as described before [62].

For protein gel blot analysis, leaf tissue from 4-week-old plants was extracted with a buffer containing 50 mM Tris-HCl, pH 7.5, 10% glycerol, 150 mM NaCl, 10 mM MgCl_2_, 5 mM EDTA, 5 mM DTT, and 1× proteinase inhibitor (Sigma). Protein concentrations were determined by the Bradford assay (Bio-Rad, CA). For immunodetection, 10–50-µg protein samples were electrophoresed on 10–15% polyacrylamide gels and run in the presence of 0.38 M Tris and 0.1% SDS. Proteins were transferred from the gels to polyvinylidene difluoride membranes by electroblotting, incubated with primary anti-HA antibody (Sigma) and alkaline phosphatase-conjugated secondary antibody (Sigma). Immunoblots were developed using color detection.

## Supporting Information

Figure S1 Relative expression levels of *R* genes in indicated genotypes. One representative quantification is shown for each Figure (noted above the graph) showing RT-PCR results. The *R* gene transcript levels were normalized for β-tubulin and relative differences in expression levels were quantified using ImageQuant TL image analysis software (GE, USA). Two-to-three independent RNA preparations were used for RT-PCR and each of these were analyzed at least twice by RT-PCR. The fold differences in expression levels were consistent between experiments and between repeats within an experiment.
(0.16 MB TIF)

Figure S2 Interaction phenotypes of *AvrRPT2* or *AvrRPS4* expressing *P. syringae* with *eds1 sid2* plants. (A) Photograph showing phenotypes produced upon infiltration of 10^5^ CFU/ml bacteria (*AvrRPT2*). The leaves were photographed at 3 days post inoculation (dpi). The mock- or pathogen-inoculated *EDS1 SID2* F2 plants showed absence of any visible symptoms in response to bacterial inoculations, similar to Col-0 or Ws-0 plants (data not shown). (B) Photograph showing phenotypes produced upon infiltration of 10^5^ CFU/mL bacteria. The leaves were photographed at 3 dpi. The phenotypes seen on pathogen inoculated *eds1-1 sid2-1* leaves were comparable to those seen on RLD (ecotype) plants, which lack a functional *RPS4* gene (data not shown). The mock- or pathogen -inoculated *EDS1 SID2* F2 plants showed absence of any visible symptoms in response to bacterial inoculations, similar to Col-0 or Ws-0 plants (data not shown).
(1.09 MB TIF)

Figure S3 Morphology, cell death, *PR-1*, and *R* gene expression and SA/SAG levels in *ssi2 eds1-2 fad7-1* and *ssi2 eds1-2 fad7-1 fad8-1* plants. (A) Comparison of the morphological phenotypes displayed by 4-week-old soil-grown wt (*SSI2*), *ssi2, ssi2 eds1, ssi2 fad7, ssi2 fad7 fad8, ssi2 eds1 fad7*, and *ssi2 eds1 fad7 fad8* plants. (B) Microscopy of trypan blue-stained leaves from indicated genotypes. (C) Expression of *PR-1* indicated genotypes. Total RNA was extracted from 4-week-old plants and used for RNA gel-blot analysis. Ethidium bromide staining of rRNA was used as loading control. (D) Endogenous SA levels in the leaves of 4-week-old plants. Values are presented as mean of three replicates and the error bars represent SD. Statistical significance was determined using Student's *t*-test. Asterisks indicate data statistically significant between *ssi2 fad7* and *ssi2 eds1 fad7* or *ssi2 fad7 fad8* and *ssi2 eds1 fad7 fad8* (P<0.05, n = 5). (E) Endogenous SAG levels in the leaves of 4-week-old plants. Values are presented as mean of three replicates and the error bars represent SD. Asterisks indicate data statistically significant between *ssi2 fad7* and *ssi2 eds1 fad7* or *ssi2 fad7 fad8* and *ssi2 eds1 fad7 fad8* (P<0.05, n = 5). (F) RT-PCR analysis of *R* genes in indicated genotypes. The level of β-tubulin was used as an internal control to normalize the amount of cDNA template. The *SSI2 EDS1 FAD7* and *SSI2 EDS1 FAD7 FAD8* F2 plants showed wt-like morphology and basal levels expression of *PR-1* and *R* genes (data not shown).
(0.96 MB TIF)

Figure S4 Morphology, cell death, *PR-1*, and *R* gene expression and SA/SAG levels in *ssi2 sag101-1, ssi2 sag101-1 eds1-2* and *ssi2 sag101-1 sid2-1* plants. (A) Comparison of the morphological phenotypes displayed by 4-week-old soil-grown wt (*SSI2*; Col-0 ecotype), *sag101, ssi2, ssi2 sag101, ssi2 sid2, ssi2 sag101 sid2, ssi2 eds1* and *ssi2 sag101 eds1* plants (scale, 0.5 cm). (B) Microscopy of trypan blue-stained leaves from indicated genotypes (scale bars, 270 microns). (C) Expression of *PR-1* in indicated genotypes. Total RNA was extracted from 3-week-old plants and used for RNA gel-blot analysis. Ethidium bromide staining of rRNA was used as the loading control. (D) RT-PCR analysis of *R* genes in indicated genotypes. The level of β-tubulin was used as an internal control to normalize the amount of cDNA template. (E) Endogenous SA levels in the leaves of 4-week-old soil-grown plants. Values are presented as averages of four replicates and the error bars represent SD. (F) Endogenous SAG levels in the leaves of 4-week-old soil-grown plants. Error bars represent SD. The *SSI2 SAG101*, *SSI2 EDS1 SAG101* and *SSI2 SAG101 SID2* F2 plants showed wt-like morphology, accumulated wt-like levels of SA and showed wt-like expression of *PR-1* and *R* genes (data not shown). Statistical significance in (E) and (F) were determined using Student's *t*-test. Asterisks indicate data statistically significant compared to results from *SSI2* (Col-0) plants (P<0.05, n = 4).
(1.17 MB TIF)

Figure S5 Morphology, cell death, *PR-1*, SA/SAG levels, and *R* gene expression in *ssi2 pad4-1 sid2-1* and *ssi2 eds5-1 sid2-1* plants. (A) Comparison of the morphological phenotypes displayed by 4-week-old soil-grown wt (*SSI2*; Col-0 ecotype), *ssi2, ssi2 sid2, ssi2 pad4, ssi2eds5, ssi2 pad4 sid2* and *ssi2 eds5 sid2* plants (scale, 0.5 cm). (B) Microscopy of trypan blue-stained leaves shown in (A) (scale bars, 270 microns). (C) Expression of *PR-1* gene in indicated genotypes. Total RNA was extracted from 4-week-old plants and used for RNA gel-blot analysis. Ethidium bromide staining of rRNA was used as the loading control. (D) RT-PCR analysis of *R* genes in indicated genotypes. The level of β-tubulin was used as an internal control to normalize the amount of cDNA template. The *SSI2 PAD4 SID2* and *SSI2 EDS5 SID2* F2 plants showed wt-like morphology and showed wt-like expression of *PR-1* and *R* genes (data not shown). (E) Endogenous SA levels in the leaves of 4-week-old soil-grown plants. Values are presented as averages of four replicates and the error bars represent SD. (F) Endogenous SAG levels in the leaves of 4-week-old soil-grown plants. Error bars represent SD. The *SSI2 PAD4* and *SSI2 EDS5* plants showed wt-like morphology, accumulated wt-like levels of SA and showed wt-like expression of *PR-1* and *R* genes (data not shown). Statistical significances in E and F were determined using Student's *t*-test. Asterisks indicate data statistically significant compared to results from *SSI2* (Col-0) plants (P<0.05, n = 4).
(1.09 MB TIF)

Table S1 A list of genetic crosses analyzed in this study.
(0.08 MB DOC)

Table S2 Fold change in transcript levels of *R* and *PR* genes in *ssi2 sid2* and *ssi2 eds1 sid2* plants compared to results from Col-0 (wt) plants. *R* genes showing 2–2.5, 2.5–3, and >3-fold activation are marked yellow, orange, or red, respectively. Transcriptional profiling was performed using Affymetrix arrays.
(0.09 MB DOC)

Table S3 FA composition from leaf tissues of *SSI2* (Col-0), *eds1, sid2, ssi2, ssi2 eds1, ssi2 sid2,* and *ssi2 eds1 sid2* plants. All measurements were made on 4-week-old plants grown at 22°C and data are described as mol%±SD calculated for a sample size of six.
(0.06 MB DOC)

Table S4 FA composition from leaf tissues of *SSI2* (Col-0), *ssi2, ssi2 eds1, fad7, ssi2 fad7, ssi2 eds1 fad7, fad7 fad8, ssi2 fad7 fad8* and *ssi2 eds1 fad7 fad8* plants. All measurements were made on plants grown at 22°C and data are described as mol%±SD calculated for a sample size of six. nd, not detected.
(0.07 MB DOC)

Table S5 Primer sequences used to amplify various genes.
(0.04 MB DOC)
