# Supplementary figures and images for: Enhanced Disease Susceptibility 1 and Salicylic Acid Act Redundantly to Regulate Resistance Gene-Mediated Signaling
Source: PLoS Genet. 2009 Jul 3;5(7):e1000545. doi: 10.1371/journal.pgen.1000545 (PMC2695777; doi:10.1371/journal.pgen.1000545)

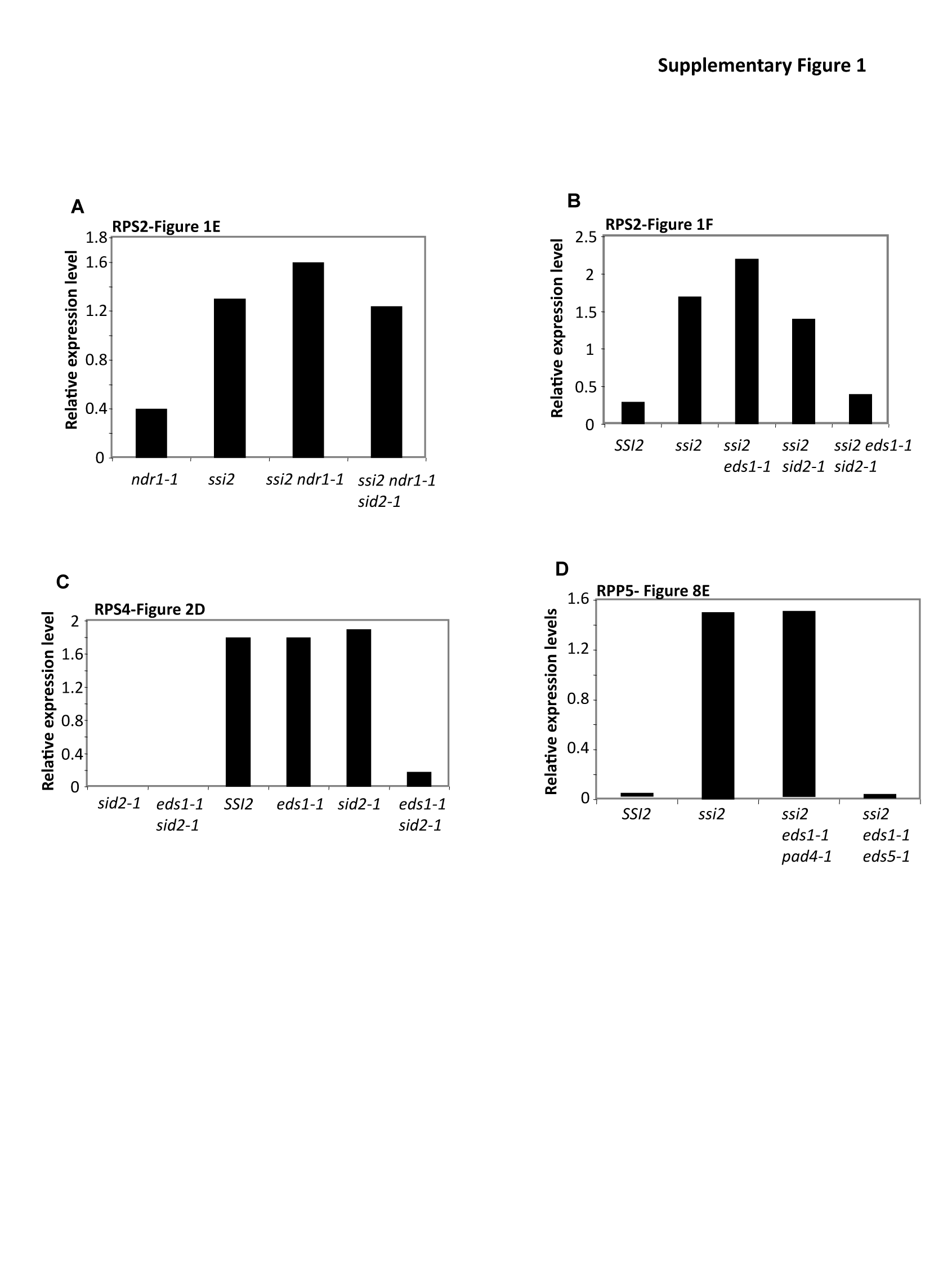

Supplement: Figure S1 — Relative expression levels of R genes in indicated genotypes. One representative quantification is shown for each Figure (noted above the graph) showing RT-PCR results. The R gene transcript levels were normalized for β-tubulin and relative differences in expression levels were quantified using ImageQuant TL image analysis software (GE, USA). Two-to-three independent RNA preparations were used for RT-PCR and each of these were analyzed at least twice by RT-PCR. The fold differences in expression levels were consistent between experiments and between repeats within an experiment. (0.16 MB TIF) [file pgen.1000545.s001.tif]

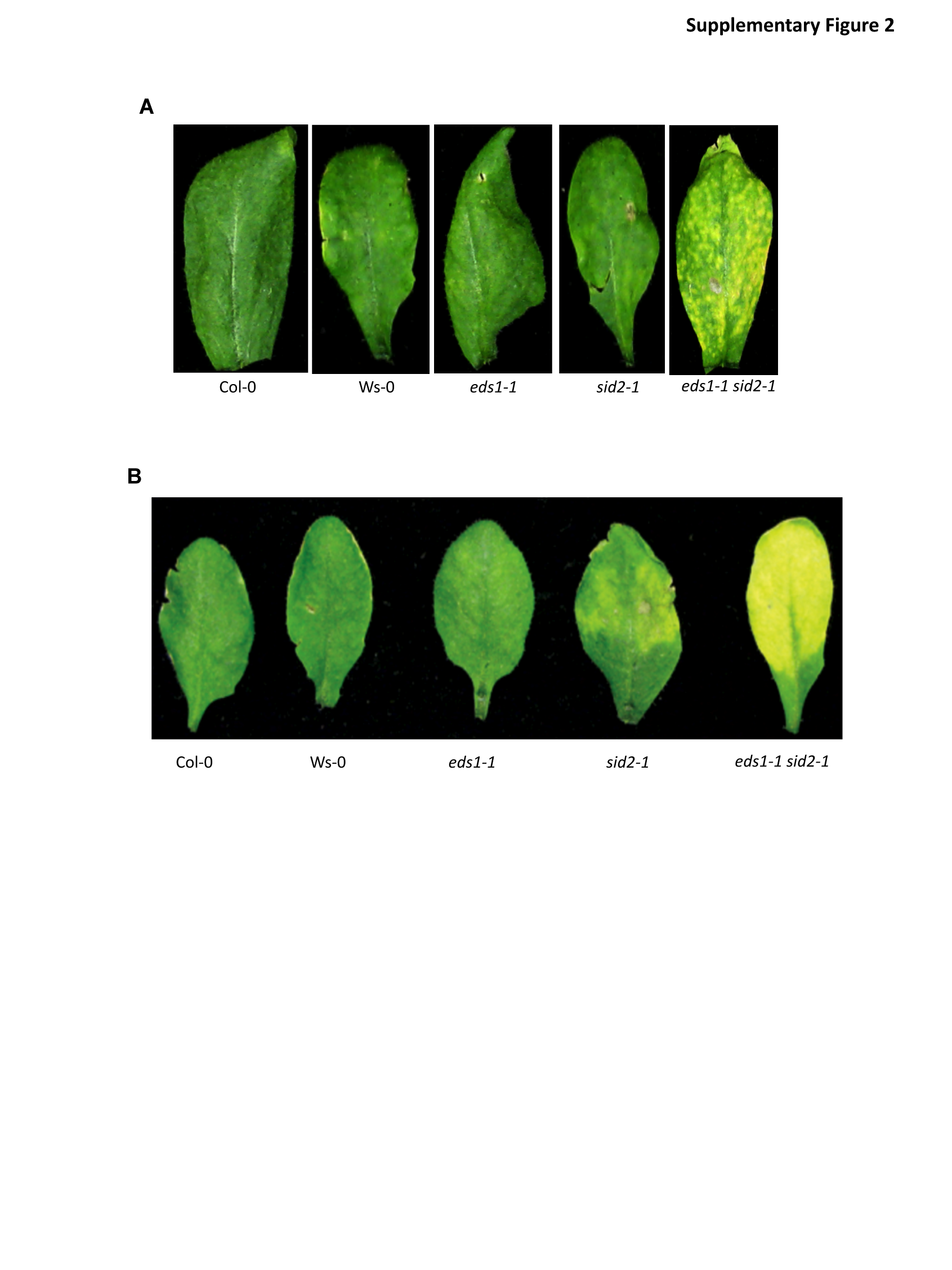

Supplement: Figure S2 — Interaction phenotypes of AvrRPT2 or AvrRPS4 expressing P. syringae with eds1 sid2 plants. (A) Photograph showing phenotypes produced upon infiltration of 105 CFU/ml bacteria (AvrRPT2). The leaves were photographed at 3 days post inoculation (dpi). The mock- or pathogen-inoculated EDS1 SID2 F2 plants showed absence of any visible symptoms in response to bacterial inoculations, similar to Col-0 or Ws-0 plants (data not shown). (B) Photograph showing phenotypes produced upon infiltration of 105 CFU/mL bacteria. The leaves were photographed at 3 dpi. The phenotypes seen on pathogen inoculated eds1-1 sid2-1 leaves were comparable to those seen on RLD (ecotype) plants, which lack a functional RPS4 gene (data not shown). The mock- or pathogen -inoculated EDS1 SID2 F2 plants showed absence of any visible symptoms in response to bacterial inoculations, similar to Col-0 or Ws-0 plants (data not shown). (1.09 MB TIF) [file pgen.1000545.s002.tif]

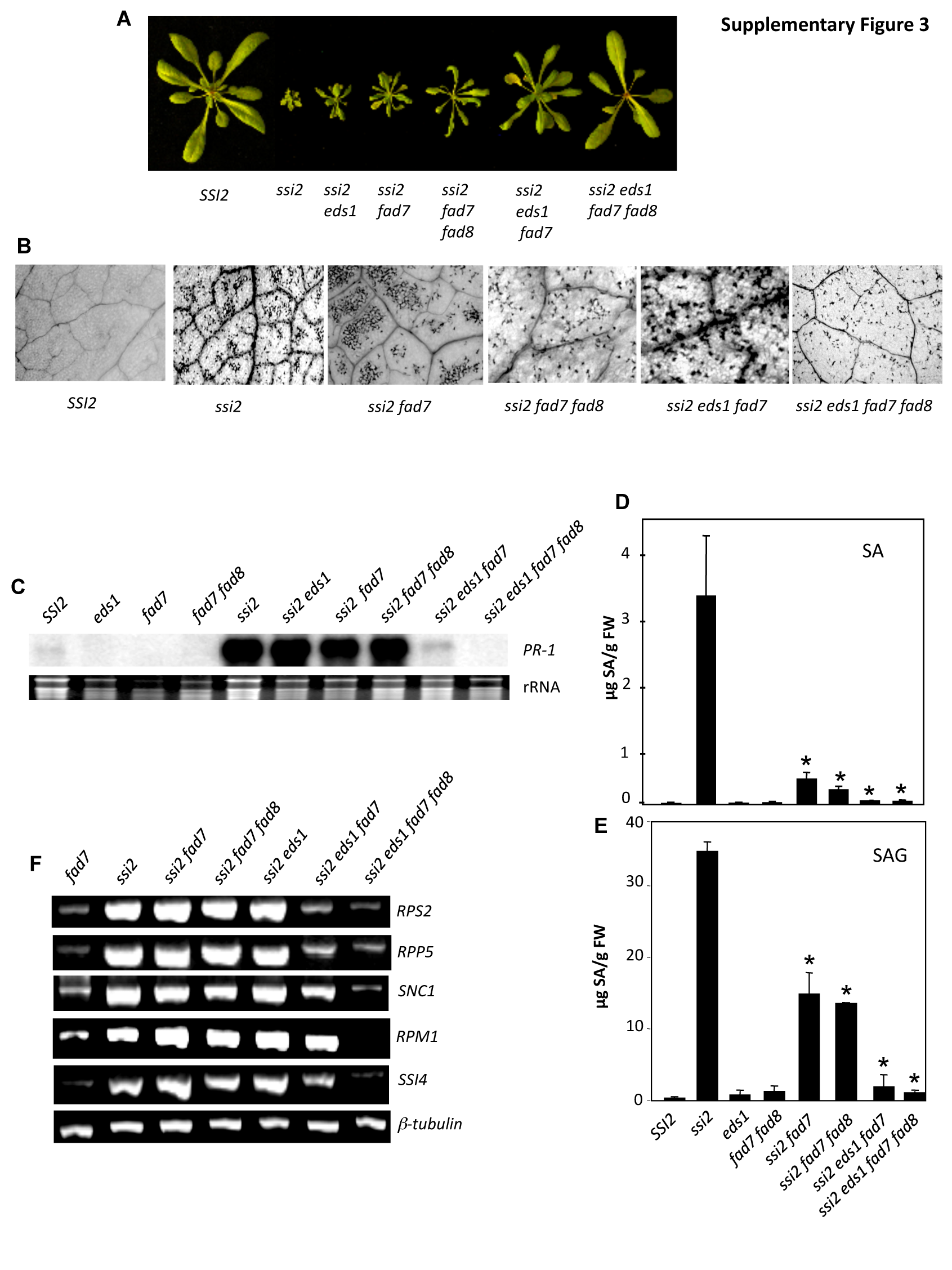

Supplement: Figure S3 — Morphology, cell death, PR-1, and R gene expression and SA/SAG levels in ssi2 eds1-2 fad7-1 and ssi2 eds1-2 fad7-1 fad8-1 plants. (A) Comparison of the morphological phenotypes displayed by 4-week-old soil-grown wt (SSI2), ssi2, ssi2 eds1, ssi2 fad7, ssi2 fad7 fad8, ssi2 eds1 fad7, and ssi2 eds1 fad7 fad8 plants. (B) Microscopy of trypan blue-stained leaves from indicated genotypes. (C) Expression of PR-1 indicated genotypes. Total RNA was extracted from 4-week-old plants and used for RNA gel-blot analysis. Ethidium bromide staining of rRNA was used as loading control. (D) Endogenous SA levels in the leaves of 4-week-old plants. Values are presented as mean of three replicates and the error bars represent SD. Statistical significance was determined using Student's t-test. Asterisks indicate data statistically significant between ssi2 fad7 and ssi2 eds1 fad7 or ssi2 fad7 fad8 and ssi2 eds1 fad7 fad8 (P<0.05, n = 5). (E) Endogenous SAG levels in the leaves of 4-week-old plants. Values are presented as mean of three replicates and the error bars represent SD. Asterisks indicate data statistically significant between ssi2 fad7 and ssi2 eds1 fad7 or ssi2 fad7 fad8 and ssi2 eds1 fad7 fad8 (P<0.05, n = 5). (F) RT-PCR analysis of R genes in indicated genotypes. The level of β-tubulin was used as an internal control to normalize the amount of cDNA template. The SSI2 EDS1 FAD7 and SSI2 EDS1 FAD7 FAD8 F2 plants showed wt-like morphology and basal levels expression of PR-1 and R genes (data not shown). (0.96 MB TIF) [file pgen.1000545.s003.tif]

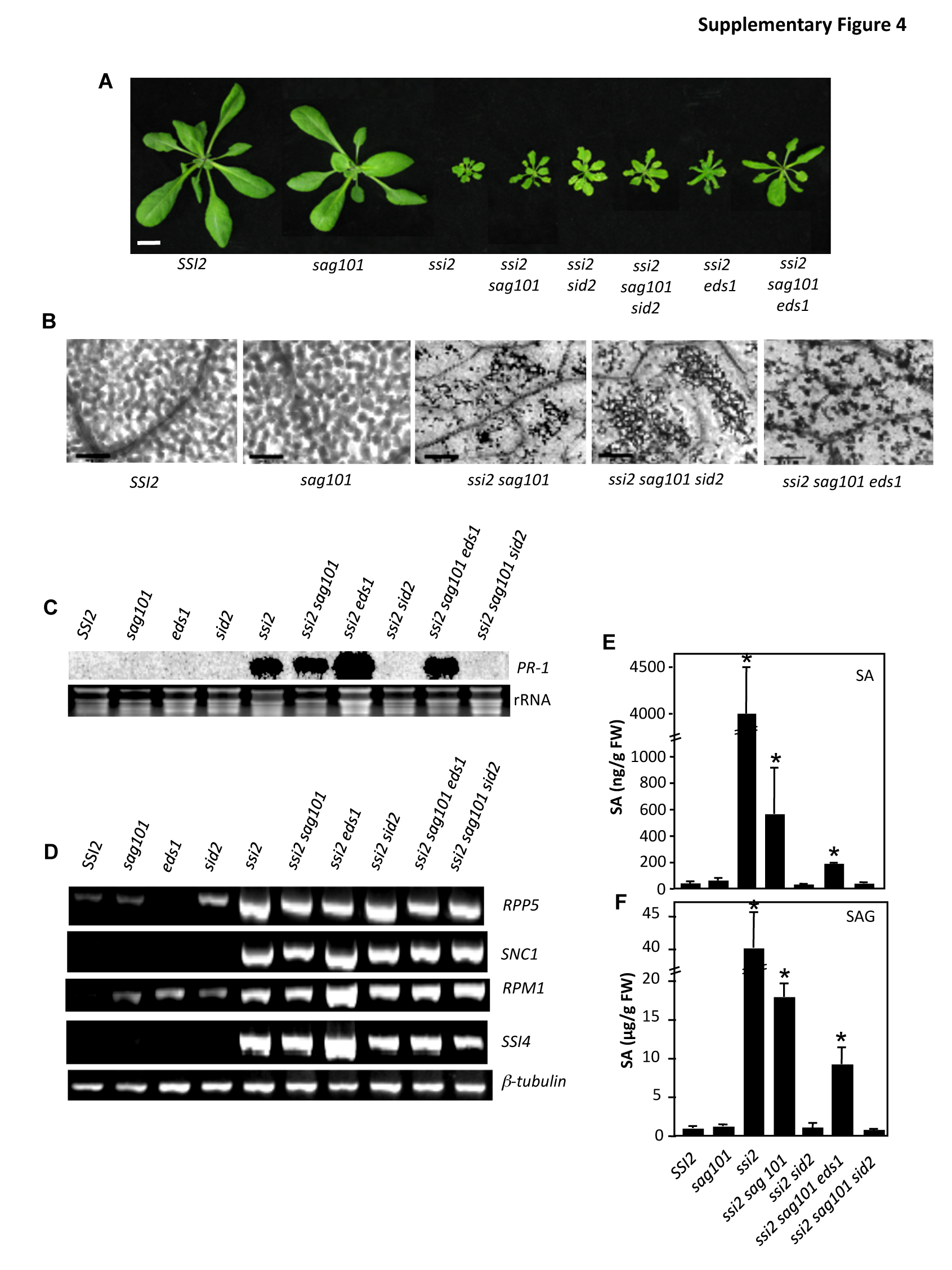

Supplement: Figure S4 — Morphology, cell death, PR-1, and R gene expression and SA/SAG levels in ssi2 sag101-1, ssi2 sag101-1 eds1-2 and ssi2 sag101-1 sid2-1 plants. (A) Comparison of the morphological phenotypes displayed by 4-week-old soil-grown wt (SSI2; Col-0 ecotype), sag101, ssi2, ssi2 sag101, ssi2 sid2, ssi2 sag101 sid2, ssi2 eds1 and ssi2 sag101 eds1 plants (scale, 0.5 cm). (B) Microscopy of trypan blue-stained leaves from indicated genotypes (scale bars, 270 microns). (C) Expression of PR-1 in indicated genotypes. Total RNA was extracted from 3-week-old plants and used for RNA gel-blot analysis. Ethidium bromide staining of rRNA was used as the loading control. (D) RT-PCR analysis of R genes in indicated genotypes. The level of β-tubulin was used as an internal control to normalize the amount of cDNA template. (E) Endogenous SA levels in the leaves of 4-week-old soil-grown plants. Values are presented as averages of four replicates and the error bars represent SD. (F) Endogenous SAG levels in the leaves of 4-week-old soil-grown plants. Error bars represent SD. The SSI2 SAG101, SSI2 EDS1 SAG101 and SSI2 SAG101 SID2 F2 plants showed wt-like morphology, accumulated wt-like levels of SA and showed wt-like expression of PR-1 and R genes (data not shown). Statistical significance in (E) and (F) were determined using Student's t-test. Asterisks indicate data statistically significant compared to results from SSI2 (Col-0) plants (P<0.05, n = 4). (1.17 MB TIF) [file pgen.1000545.s004.tif]

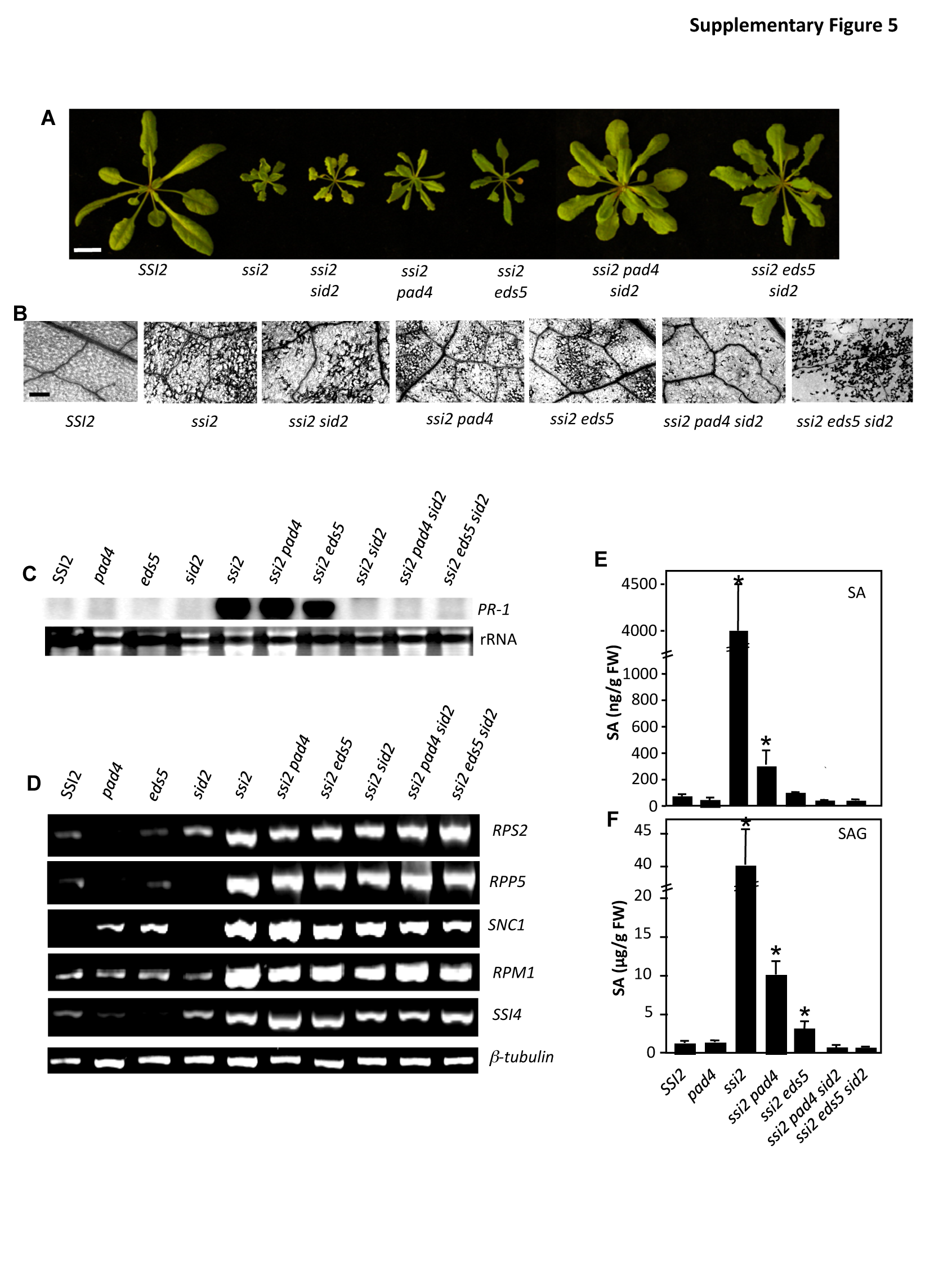

Supplement: Figure S5 — Morphology, cell death, PR-1, SA/SAG levels, and R gene expression in ssi2 pad4-1 sid2-1 and ssi2 eds5-1 sid2-1 plants. (A) Comparison of the morphological phenotypes displayed by 4-week-old soil-grown wt (SSI2; Col-0 ecotype), ssi2, ssi2 sid2, ssi2 pad4, ssi2eds5, ssi2 pad4 sid2 and ssi2 eds5 sid2 plants (scale, 0.5 cm). (B) Microscopy of trypan blue-stained leaves shown in (A) (scale bars, 270 microns). (C) Expression of PR-1 gene in indicated genotypes. Total RNA was extracted from 4-week-old plants and used for RNA gel-blot analysis. Ethidium bromide staining of rRNA was used as the loading control. (D) RT-PCR analysis of R genes in indicated genotypes. The level of β-tubulin was used as an internal control to normalize the amount of cDNA template. The SSI2 PAD4 SID2 and SSI2 EDS5 SID2 F2 plants showed wt-like morphology and showed wt-like expression of PR-1 and R genes (data not shown). (E) Endogenous SA levels in the leaves of 4-week-old soil-grown plants. Values are presented as averages of four replicates and the error bars represent SD. (F) Endogenous SAG levels in the leaves of 4-week-old soil-grown plants. Error bars represent SD. The SSI2 PAD4 and SSI2 EDS5 plants showed wt-like morphology, accumulated wt-like levels of SA and showed wt-like expression of PR-1 and R genes (data not shown). Statistical significances in E and F were determined using Student's t-test. Asterisks indicate data statistically significant compared to results from SSI2 (Col-0) plants (P<0.05, n = 4). (1.09 MB TIF) [file pgen.1000545.s005.tif]
