# Supplementary material for: Enhanced Disease Susceptibility 1 and Salicylic Acid Act Redundantly to Regulate Resistance Gene-Mediated Signaling
Source: PLoS Genet. 2009 Jul 3;5(7):e1000545. doi: 10.1371/journal.pgen.1000545 (PMC2695777; doi:10.1371/journal.pgen.1000545)
Supplement: Table S1 — A list of genetic crosses analyzed in this study. (0.08 MB DOC) [file pgen.1000545.s006.doc]

**Supplemental Table 1.** A list of genetic crosses analyzed in this study.

| **Genotypes created** | **Crosses analyzed** | **Phenotype^a, b, c, d, e, f, g^** |
| --- | --- | --- |
| *ssi2 ndr1* | *ssi2* (Nössen ecotype) x *ndr1-1* (Col-0 background) | 15 *ssi2 ndr1* plants were analyzed**^h^** in the F2 generation, 4 were selfed and reanalyzed in F3 and F4 generations. |
| *ssi2 sid2* | *ssi2* x *sid2-1* (Col-0 background) | 17 *ssi2 sid2* plants were analyzed in the F2 generation, 5 were selfed and reanalyzed in F3 and F4 generations. |
| *ssi2 ndr1 sid2* | *ssi2 ndr1-1* x *ssi2 sid2-1*  *ssi2 ndr1-1* x *sid2-1* | 11 *ssi2 ndr1 sid2* plants were analyzed in the F2 generation, 3 were selfed and reanalyzed in F3 and F4 generations. |
| *ssi2 eds1* | *ssi2* x *eds1-1* (Ws ecotype)  *ssi2* x *eds1-2* (L*er* ecotype) | 11 *ssi2 eds1-1* and 13 *ssi2 eds1-2* plants were analyzed in F2 generation, 3 lines from each F2 population were selfed and reanalyzed in F3 and F4 generations. |
| *ssi2 eds1 sid2* | *ssi2 eds1-1* x *sid2-1*  *ssi2 sid2-1* x *eds1-1*  *ssi2 eds1-2* x *sid2-1*  *ssi2 sid2-1* x *eds1-2* | ~11 *ssi2 eds1 sid2* plants from each cross were analyzed in F2 generation, 4 F2 plants from each cross were selfed and reanalyzed in the F3 and F4 generations. |
| *ssi2 eds1 nahG* | *ssi2 eds1-1* x Nössen-NahG  *ssi2 eds1-2* x Nössen-NahG | 13 *ssi2 eds1 nahG* plants were analyzed in F2 generation, 4 were selfed and reanalyzed in the F3 and F4 generations. |
| *eds1 sid2* | *eds1-1* x *sid2-1*  *eds1-2* x *sid2-1*  *ssi2 eds1-1* x *sid2-1*  *ssi2 eds1-2* x *sid2-1* | ~12 F2’s from each cross were analyzed, 6 were selfed and reanalyzed in the F3 generation.  *EDS1 SID2* F2 plants derived from these crosses were used as controls in all experiments and showed wt-like phenotypes. |
| *eds1-2 nahG* | *eds1-2* (Ler background) x Ler-NahG | The F2’s were scored for plants homozygous for *eds1-2* and containing at least one copy of NahG. Only *eds1-2* plants containing at least one copy of NahG developed infection. Five such lines were tested for pathogen infections in F3 and F4 generations. All pathogen- inoculated plants were confirmed by genotyping for *eds1-2* and presence of NahG transgene. |
| *ssi2 eds5* | *ssi2* x *eds5-1* (Col-0 background). | 14 *ssi2 eds5* F2 plants were analyzed, 4 F2 plants were selfed and reanalyzed in F3 and F4 generations. |
| *ssi2 eds5 sid2* | *ssi2 sid2-1* x *ssi2 eds5-1*  *ssi2 eds5-1* x *sid2-1* | A total of 16 *ssi2 eds5 sid2* F2 plants were analyzed from both crosses, 5 were selfed and reanalyzed in F3 and F4 generations. |
| *ssi2 eds1 eds5* | *ssi2 eds1-2* x *ssi2 eds5-1*  *ssi2 eds1-2* x *eds5-1* | 7 *ssi2 eds1 eds5* F2 plants were analyzed from each cross, all of these were selfed and reanalyzed in F3 and F4 generations. |
| *ssi2 eds5 nahG* | *ssi2 eds5-1* x Nössen-NahG | 8 *ssi2 eds5 nahG* F2 plants were analyzed, 4 selfed and reanalyzed in F3 and F4 generations. |
| *ssi2 pad4* | *ssi2* x *pad4-1* (Col-0 background) | 16 *ssi2 pad4* F2 plants were analyzed, 4 F2 plants were selfed and reanalyzed in F3 and F4 generations. |
| *ssi2 eds1 pad4* | *ssi2 eds1-2* x *ssi2 pad4-1*  *ssi2 eds1-1* x *pad4-1* | Since *PAD4* and *EDS1* are located within 10 cM of each other, a total of 4 *ssi2 eds1 pad4* F2 plants were obtained from these two crosses. These were selfed and reanalyzed in F3 and F4 generations. |
| *ssi2 pad4 sid2* | *ssi2 pad4-1* x *ssi2 sid2-1*  *ssi2 pad4-1* x *sid2-1* | 8 *ssi2 pad4 sid2* F2 plants from each cross were analyzed, all were selfed and reanalyzed in F3 and F4 generations. |
| *ssi2 pad4 nahG* | *ssi2 pad4-1* x Nössen-NahG | 8 *ssi2 pad4 nahG* F2 plants were analyzed, selfed and reanalyzed in F3 and F4 generations. |
| *ssi2 sag101* | *ssi2* x *sag101-1* (Col-0 background) | 8 *ssi2 sag101* plants were analyzed in F2 generation, 2 were selfed and reanalyzed in F3 and F4 generations. |
| *ssi2 sag101 sid2* | *ssi2 sag101-1* x *sid2-1*  *ssi2 sag101-1* x *ssi2 sid2-1* | 19 *ssi2 sag101 sid2* plants were analyzed in F2 generation, 4 were selfed and reanalyzed in F3 and F4 generations. |
| *HRT sid2* | Di-17 (Dijon ecotype) x *sid2-1* | 12 *HRT sid2* plants were analyzed in F2 generation, 4 were selfed and reanalyzed in F3 and F4 generations. |
| *ssi2 fad7* | *ssi2* x *fad7-1* (Col-0 background) | 12 plants were analyzed in F2 generation, all were selfed and reanalyzed in F3 and F4 generations. |
| *ssi2 eds1 fad7* | *ssi2 eds1-2* x *ssi2 fad7-1* | 7 *ssi2 eds1 fad7* plants were analyzed in F2 generation, 4 of these were selfed and reanalyzed in F3 and F4 generation. |
| *ssi2 fad7 fad8* | *ssi2* x *fad7-1 fad8-1* double mutant (Col-0 background). | 8 *ssi2 fad7 fad8* plants were analyzed in F2 generation, 4 of these were selfed and reanalyzed in F3 and F4 generation. |
| *ssi2 eds1 fad7 fad8* | *ssi2 eds1-2* x *ssi2 fad7-1 fad8-1* | 4 *ssi2 eds1 fad7 fad8* plants were analyzed in F2 generation, all of these were selfed and reanalyzed in F3 and F4 generation. |
| *HRT eds1* | Di-17 x *eds1-1* | 15 *HRT eds1* plants were analyzed in F2 generation, 4 were selfed and reanalyzed in F3 and F4 generations. |
| *HRT eds1 sid2* | *HRT eds1-1* x *HRT sid2-1*  Di-17 x *eds1-1 sid2-1* | 11 *HRT eds1 sid2* plants were analyzed in F2 generation, 6 were selfed and reanalyzed in F3 and F4 generations. |
| *HRT ssi2* | Di-17 x *ssi2* | 21 *HRT ssi2* plants were analyzed in F2 generation, 4 were selfed and reanalyzed in F3 and F4 generations. |
| *HRT ssi2 eds1* | Di-17 x *ssi2 eds1-1*  *HRT eds1-1* x *ssi2 eds1-1* | 25 *HRT ssi2 eds1* plants were analyzed in F2 generation, 3 were selfed and reanalyzed in F3 and F4 generations. |
| *HRT ssi2 sid2* | Di-17 x *ssi2 sid2-1*  *HRT sid2-1* x *ssi2 sid2-1*  *HRT ssi2* x *sid2-1* | 32 *HRT ssi2 sid2* plants were analyzed in F2 generation, 4 were selfed and reanalyzed in F3 and F4 generations. |
| *HRT ssi2 eds1 sid2* | Di-17 x *ssi2 eds1-1 sid2-1*  *HRT ssi2* x *ssi2 eds1-1 sid2-1*  *HRT sid2-1* x *ssi2 eds1-1* | 27 *HRT ssi2 eds1 sid2* plants were analyzed in F2 generation, 4 were selfed and reanalyzed in F3 and F4 generations. |

^a^ Unless otherwise mentioned, all single, double or triple mutant plants showed similar morphological phenoypes.

^b^ Unless otherwise mentioned, different alleles segregated in a Mendelian fashion.

^c^  Analyses carried out in the F2 generation included FA profiling, *PR* gene expression, staining to visualize cell death, and pathogen resistance.

^d^  About 30-40 F3 plants were analyzed in F3 generations, two plants from F3’s were selfed and reexamined in F4 generation. Plants were evaluated for morphological phenotype, FA profiling, *PR* gene expression, staining to visualize cell death, and pathogen resistance.

^e^  F2 plants that contained wild-type alleles were used as controls in all experiments. These behaved similarly to wild-type parents for morphological phenotype, cell death, FA profile, *PR* gene expression and pathogen resistance.

^f^  In addition to the crosses mentioned in this Table, control crosses between *ssi2* x Col-0, *ssi2* x L*er*, *ssi2* x Ws, Di-17 x No, Di-17 x Col-0, Di-17 x L*er*, Di-17 x Ws were also studied. All crosses involving *ssi2* segregated for *ssi2* in a Mendelian fashion (3 wild-type plants to 1 *ssi2*). The *SSI2* allele segregated in 1:2:1 manner. All control crosses involving TCV resistant ecotype, Di-17, segregated resistance and HR in an expected manner; HR cosegregated with *HRT* (*R* gene conferring HR to TCV) and resistance cosegregated with *HRT* and *rrt* (in a recessive manner; Chandra-Shekara et al, 2004).

^g^ Genotypes were confirmed by PCR, CAPS, dCAPS analysis and/or FA profiling.

^h^ Indicates number of plants analyzed at molecular or biochemical level. At least three times more plants were analyzed at the morphological level.
