# Supplementary material for: Enhanced Disease Susceptibility 1 and Salicylic Acid Act Redundantly to Regulate Resistance Gene-Mediated Signaling
Source: PLoS Genet. 2009 Jul 3;5(7):e1000545. doi: 10.1371/journal.pgen.1000545 (PMC2695777; doi:10.1371/journal.pgen.1000545)
Supplement: Table S2 — Fold change in transcript levels of R and PR genes in ssi2 sid2 and ssi2 eds1 sid2 plants compared to results from Col-0 (wt) plants. R genes showing 2–2.5, 2.5–3, and >3-fold activation are marked yellow, orange, or red, respectively. Transcriptional profiling was performed using Affymetrix arrays. (0.09 MB DOC) [file pgen.1000545.s007.doc]

**Supplemental Table 2.** Fold change in transcript levels of *R* and *PR* genes in *ssi2 sid2* and *ssi2 eds1 sid2* plants compared to results from Col-0 (wt) plants. *R* genes showing 2-2.5, 2.5-3 and >3-fold activation are marked yellow, orange or red, respectively. Transcriptional profiling was performed using Affymetrix arrays.

| **S. No.** | **Overall_F_Pvalue** | **AGI_No.** | ***ssi2 sid2*/Col-0** | ***ssi2 eds1 sid2/* Col-0** |
| --- | --- | --- | --- | --- |
| 1 | 0.000414414 | AT1G72940 | 9.193292683 | 2.39054878 |
| 2 | 9.97222E-05 | AT1G66090 | 6.007416564 | 2.47342398 |
| 3 | 0.011366274 | AT4G16890 (SNC1) | 5.938187702 | 1.439158576 |
| 4 | 2.30029E-07 | AT1G17600 | 4.725274725 | 1.232600733 |
| 5 | 0.000410926 | AT1G12280 | 4.336214347 | 2.513396716 |
| 6 | 9.15809E-05 | AT1G72890 | 4.159223755 | 1.256254384 |
| 7 | 0.006451513 | AT5G46450 | 4.150770512 | 1.119533528 |
| 8 | 0.00394764 | AT4G16860 | 4.025021949 | 1.262510975 |
| 9 | 0.002601929 | AT3G46530 | 3.670633532 | 1.368043402 |
| 10 | 0.000145129 | AT1G72900 | 3.662160406 | 1.032237099 |
| 11 | 1.36967E-05 | AT1G61180 | 3.198901939 | 0.970672041 |
| 12 | 0.000224136 | AT5G46260 | 2.995641646 | 1.101210654 |
| 13 | 0.02199773 | AT1G33560 | 2.506788447 | 1.277709208 |
| 14 | 1.89092E-05 | AT4G19500 | 2.417882645 | 1.757063024 |
| 15 | 1.89092E-05 | AT4G19500 | 2.417882645 | 1.757063024 |
| 16 | 0.040991407 | AT3G50950 | 2.252965645 | 1.014841062 |
| 17 | 9.52298E-05 | AT4G19510 | 2.243735763 | 2.106150342 |
| 18 | 0.026401949 | AT1G53350 | 2.162810626 | 0.914738646 |
| 19 | 0.000166608 | AT1G63880 | 2.136663981 | 1.285898469 |
| 20 | 5.99486E-05 | AT4G33300 | 2.104964718 | 1.141507056 |
| 21 | 2.5881E-08 | AT1G69550 | 2.027997565 | 0.940353013 |
| 22 | 0.055799435 | AT3G07040 (RPM1) | 1.848037999 | 0.948402482 |
| 23 | 0.052922722 | AT4G26090 (RPS2) | 1.625628141 | 0.494556114 |
| 24 | 0.087059995 | AT4G16950 (RPP5) | 1.654512472 | 0.539229025 |
| 25 | 0.080579787 | AT5G45250 (RPS4) | 1.46181503 | 0.521200099 |
| 26 | 1.36618E-05 | AT2G14610 (PR-1) | 0.259355897 | 0.877217832 |
| 27 | 4.01491E-05 | AT3G57260 (PR-2) | 25.65704438 | 0.877217832 |
| 28 | 0.00081914 | AT1G75040 (PR-5) | 30.41574458 | 4.544029507 |
