# Supplementary material for: Enhanced Disease Susceptibility 1 and Salicylic Acid Act Redundantly to Regulate Resistance Gene-Mediated Signaling
Source: PLoS Genet. 2009 Jul 3;5(7):e1000545. doi: 10.1371/journal.pgen.1000545 (PMC2695777; doi:10.1371/journal.pgen.1000545)
Supplement: Table S3 — FA composition from leaf tissues of SSI2 (Col-0), eds1, sid2, ssi2, ssi2 eds1, ssi2 sid2, and ssi2 eds1 sid2 plants. All measurements were made on 4-week-old plants grown at 22°C and data are described as mol%±SD calculated for a sample size of six. (0.06 MB DOC) [file pgen.1000545.s008.doc]

**Supplemental Table 3.** FA composition from leaf tissues of *SSI2* (Col-0), *eds1*, *sid2*, *ssi2*, *ssi2* *eds1*, *ssi2* *sid2,* and *ssi2* *eds1 sid2* plants. All measurements were made on 4-week-old plants grown at 22°C and data are described as mol% ± SD calculated for a sample size of six.

| **Genotype** | **16:0** | **16:1** | **16:2** | **16:3** | **18:0** | **18:1** | **18:2** | **18:3** |
| --- | --- | --- | --- | --- | --- | --- | --- | --- |
| ***SSI2*** | 16.8 ± 1.3 | 4.8 ± 0.8 | 0.6 ± 0.0 | 15.3 ± 1.2 | 0.9 ± 0.4 | 2.6 ± 0.2 | 15.3 ± 0.9 | 43.7 ± 3.1 |
| ***eds1*** | 16.1 ± 0.7 | 4.5 ± 0.1 | 0.6 ± 0.3 | 18.2 ± 1.7 | 0.5 ± 0.3 | 1.8 ± 0.2 | 13.8 ± 1.3 | 44.5 ± 1.3 |
| ***sid2*** | 15.4 ± 0.3 | 4.1 ± 0.4 | 0.5 ± 0.1 | 17.6 ± 1.6 | 0.3 ± 0.0 | 2.2 ± 0.7 | 12.4 ± 1.2 | 47.5 ± 0.6 |
| ***ssi2*** | 16.5 ± 1.5 | 3.5 ± 0.5 | 0.5 ± 0.2 | 10.7 ± 0.2 | 12.9 ± 1.4 | 0.6 ± 0.1 | 10.3 ± 0.9 | 45.0 ± 1.2 |
| ***ssi2 eds1*** | 15.9 ± 2.3 | 3.3 ± 0.4 | 0.7 ± 0.0 | 13.7± 1.2 | 13.6 ± 1.4 | 0.9 ± 0.2 | 10.3 ± 0.3 | 41.6 ± 3.3 |
| ***ssi2 sid2*** | 18.9 ± 2.7 | 4.0 ± 0.2 | 0.6 ± 0.0 | 12.2 ± 0.6 | 11.2 ± 0.6 | 0.5 ± 0.1 | 8.9 ± 0.8 | 43.7 ± 3.1 |
| ***ssi2 eds1 sid2*** | 18.0 ± 1.4 | 3.5 ± 0.3 | 0.5 ± 0.0 | 12.7 ± 0.9 | 11.3 ± 2.8 | 0.6 ± 0.1 | 9.7 ± 0.9 | 43.7 ± 3.2 |
