# Supplementary material for: Enhanced Disease Susceptibility 1 and Salicylic Acid Act Redundantly to Regulate Resistance Gene-Mediated Signaling
Source: PLoS Genet. 2009 Jul 3;5(7):e1000545. doi: 10.1371/journal.pgen.1000545 (PMC2695777; doi:10.1371/journal.pgen.1000545)
Supplement: Table S4 — FA composition from leaf tissues of SSI2 (Col-0), ssi2, ssi2 eds1, fad7, ssi2 fad7, ssi2 eds1 fad7, fad7 fad8, ssi2 fad7 fad8 and ssi2 eds1 fad7 fad8 plants. All measurements were made on plants grown at 22°C and data are described as mol%±SD calculated for a sample size of six. nd, not detected. (0.07 MB DOC) [file pgen.1000545.s009.doc]

***Supplemental Table 4.*** *FA composition from leaf tissues of SSI2 (Col-0), ssi2, ssi2 eds1, fad7, ssi2 fad7, ssi2 eds1 fad7, fad7 fad8, ssi2 fad7 fad8 and ssi2 eds1 fad7 fad8 plants. All measurements were made on plants grown at 22°C and data are described as mol% ± SD calculated for a sample size of six. nd, not detected.*

| Genotype | 16:0 | 16:1 | 16:2 | 16:3 | 18:0 | 18:1 | 18:2 | 18:3 |
| --- | --- | --- | --- | --- | --- | --- | --- | --- |
| *SSI2* | 17.8 ± 4.9 | 4.1 ± 0.6 | 0.6 ± 0.5 | 16.7 ± 1.8 | 0.6 ± 0.2 | 2.6 ± 0.6 | 13.7 ± 1.7 | 43.9 ± 4.0 |
| *ssi2* | 14.6 ± 1.3 | 3.8 ± 0.6 | 0.4 ± 0.2 | 10.2 ± 5.4 | 14.5 ± 2.6 | 0.8 ± 0.4 | 15.0 ± 7.6 | 40.7 ± 3.9 |
| *ssi2 eds1* | 18.2 ± 0.9 | 3.3 ± 0.4 | 0.5 ± 0.1 | 12.2 ± 1.7 | 15.8 ± 2.8 | 0.8 ± 0.1 | 9.7 ± 0.4 | 39.5 ± 2.1 |
| *fad7* | 14.4 ± 2.0 | 4.8 ± 1.3 | 6.5 ±2.5 | 4.3 ± 1.1 | 0.7 ± 0.2 | 3.2 ± 0.6 | 34.5 ± 6.9 | 31.6 ± 4.3 |
| *ssi2 fad7* | 12.7 ± 1.2 | 3.2 ± 0.8 | 9.8 ± 0.7 | 4.1 ± 1.0 | 16.3 ± 1.8 | 1.9 ± 0.2 | 19.8 ± 0.7 | 32.3 ± 1.3 |
| *ssi2 eds1 fad7* | 12.7 ± 1.7 | 4.2 ± 0.2 | 7.0 ± 0.6 | 6.7 ± 1.6 | 16.0 ± 1.5 | 1.8 ± 0.9 | 18.9 ± 1.2 | 32.7 ± 5.2 |
| *fad7 fad8* | 14.3 ± 2.8 | 5.0 ± 0.4 | 11.9 ± 6.3 | 0.2 ± 0.5 | 0.7 ± 0.1 | 3.7 ± 0.8 | 53.7 ± 3.0 | 10.5 ± 1.1 |
| *ssi2 fad7 fad8* | 10.3 ± 0.8 | 4.1 ± 0.2 | 13.0 ± 0.9 | nd | 15.5 ± 1.7 | 3.0 ± 0.2 | 37.0 ± 3.5 | 17.1 ± 2.7 |
| *ssi2 eds1 fad7 fad8* | 10.0 ± 1.0 | 4.7 ± 0.4 | 17.5 ± 0.9 | nd | 11.2 ± 0.9 | 2.1 ± 0.3 | 44.2 ± 2.5 | 10.3 ± 1.4 |
