# Supplementary material for: Enhanced Disease Susceptibility 1 and Salicylic Acid Act Redundantly to Regulate Resistance Gene-Mediated Signaling
Source: PLoS Genet. 2009 Jul 3;5(7):e1000545. doi: 10.1371/journal.pgen.1000545 (PMC2695777; doi:10.1371/journal.pgen.1000545)
Supplement: Table S5 — Primer sequences used to amplify various genes. (0.04 MB DOC) [file pgen.1000545.s010.doc]

**Supplemental Table 5.** Primer sequences used to amplify various genes.

| ORF | Primers | |
| --- | --- | --- |
| *SSI4* | CTCAAGAGAGTATGCTTCTCTTTCCATAACCC  CTGGTTTGGTCTTCATGAGACTCCATGAG | |
| *RPS2* | ATGGATTTCATCTCATCTCTT  TATAATCTCCGCGAGCCGGCG | |
| *RPS4* | ATGGAGACATCATCTATTTCCACTG  AATTCCGGGCATCCCAACAACTCCA | |
| *RPM1* | GCATACATGGGACCTAGGTTGCGTTTTGCACAAGG  GCCTTGGCCGCCTAAGATGAGAGGCTCAC | |
| *RPP5* | ATGGCGGCTTCTTCTTCTTCT  CCCAAAAGCAGATCGGCATAA | |
| *SNC1* | ATGGAGATAGCTTCTTCTTCT  ATCAGGTGGAGAGTCTTTCCC | |
| *RPP28* | ATGGATTTTTACGTTTTCCT  CATGGCAGCTTCGGTATCCC | |
| *HRT* | CATTTCCCTACCTCTAATGGATGA  ATGACTTTTGTGAAGCAGCCTCTA | |
| *RPP8* | CCACTTGAGATACTTGAGTTTATTTT  CCAGCGAAAGCCTTAAATGTTAATT | |
| *PR-2* | GATGGATGTTGGCCGTGTCT  CTTCCTCGTGTTCATCACAAG | |
| *β-tubulin* | | CGTGGATCACAGCAATACAGAGCC  CCTCCTGCACTTCCACTTCGTCTT C |
